# Mutations in tyrosyl-DNA phosphodiesterase 2 suppress *top-2* induced chromosome segregation defects during *Caenorhabditis elegans* spermatogenesis

**DOI:** 10.1016/j.jbc.2024.107446

**Published:** 2024-06-04

**Authors:** Ji Kent Kwah, Nirajan Bhandari, Christine Rourke, Gabriella Gassaway, Aimee Jaramillo-Lambert

**Affiliations:** Department of Biological Sciences, University of Delaware, Newark, Delaware, USA

**Keywords:** *topoisomerase II*, TOP-2, *tdp2*, TDPT-1, chromosome structure, *C. elegans*, meiosis

## Abstract

Meiosis reduces ploidy through two rounds of chromosome segregation preceded by one round of DNA replication. In meiosis I, homologous chromosomes segregate, while in meiosis II, sister chromatids separate from each other. Topoisomerase II (Topo II) is a conserved enzyme that alters DNA structure by introducing transient double-strand breaks. During mitosis, Topo II relieves topological stress associated with unwinding DNA during replication, recombination, and sister chromatid segregation. Topo II also plays a role in maintaining mitotic chromosome structure. However, the role and regulation of Topo II during meiosis is not well-defined. Previously, we found an allele of Topo II, *top-2(it7)*, disrupts homologous chromosome segregation during meiosis I of *Caenorhabditis elegans* spermatogenesis. In a genetic screen, we identified different point mutations in 5′-tyrosyl-DNA phosphodiesterase two (Tdp2, *C. elegans tdpt-1*) that suppress *top-2(it7)* embryonic lethality. Tdp2 removes trapped Top-2-DNA complexes. The *tdpt-1* suppressing mutations rescue embryonic lethality, ameliorate chromosome segregation defects, and restore TOP-2 protein levels of *top-2(it7)*. Here, we show that both TOP-2 and TDPT-1 are expressed in germ line nuclei but occupy different compartments until late meiotic prophase. We also demonstrate that *tdpt-1* suppression is due to loss of function of the protein and that the *tdpt-1* mutations do not have a phenotype independent of *top-2(it7)* in meiosis. Lastly, we found that the *tdpt-1* suppressing mutations either impair the phosphodiesterase activity, affect the stability of TDPT-1, or disrupt protein interactions. This suggests that the WT TDPT-1 protein is inhibiting chromosome biological functions of an impaired TOP-2 during meiosis.

During sexual reproduction, diploid organisms have a set of cells that undergo meiosis to generate haploid gametes that when united at fertilization maintains genomic continuity between generations. Cells in meiosis undergo two nuclear divisions preceded by a single S phase. In the first meiotic division, homologous chromosomes separate, which requires the coordination of homologous chromosome pairing, synapsis, recombination, chromosome remodeling, and the targeted loss of sister chromatid cohesion from specific chromosome domains. These events must be tightly organized and executed to promote the accurate segregation of homologous chromosomes during meiosis I, followed by the separation of sister chromatids during the second meiotic division.

DNA topoisomerase II (Topo II) is an essential enzyme that is required to relieve topological stress associated with the unwinding of DNA during replication, recombination, and chromosome segregation as well as the maintenance of chromosome structure during the mitotic cell cycle (1). Topo II is an ATP-dependent, homodimeric type II topoisomerase which introduces double-strand breaks (DSBs) into DNA. Vertebrates have two isozymes of Topo II: TOP2α and TOP2β, that differ in their C-terminal domains (2). *Caenorhabditis elegans* has a single Topo II homolog (TOP-2), which shares 52% amino-acid sequence identity with human TOP2α (3). Topo II catalyzes the passage of one segment of duplex DNA through another by utilizing a reversible transesterification reaction (1, 4, 5). Topo II captures one of the DNA duplexes and then introduces a DSB to open a gate in the segment of DNA. During the creation of a DSB, the Topo II active site tyrosine forms a covalent adduct with the DNA. This results in a cleavage complex containing a DSB with a four base pair overhang in which the active site tyrosine is covalently connected to the 5′ phosphate of the DNA backbone. The Topo II cleavage complex allows for a second DNA duplex to be transported through the gate. This phenomenon alters the topology of the two DNA duplexes. Resealing of the DSB is carried out *via* a second transesterification reaction freeing the two catalytic tyrosines of the Topo II homodimers and ligating the broken DNA strands back together (1, 4, 5).

During meiosis, Topo II is associated with chromosomes and, in several organisms, is required for the accurate segregation of homologous chromosomes at meiosis I (6, 7, 8, 9, 10, 11). In *C. elegans*, TOP-2 is expressed throughout meiosis of both spermatogenesis and oogenesis; however, analysis of a temperature sensitive, loss-of-function allele of *top-2* [*top-2(it7)*] suggests a spermatogenesis-specific requirement for TOP-2 to accurately segregate homologous chromosomes at meiosis I (3). Previous characterization of *top-2(it7)* found that sperm that develop at the restrictive temperature of 24 °C have chromosome segregation defects at anaphase I of meiosis, which results in embryonic lethality after fertilization (3). *top-2(it7)* is a missense allele that changes arginine 828 to cysteine. This allele results in mislocalization of TOP-2 in the germline; TOP-2 fails to associate with the paired homologous chromosomes throughout meiotic prophase. In addition, protein expression is slightly reduced in the *top-2* [R828C] mutants (3). While TOP-2 is expressed and required for meiotic processes, how TOP-2 is regulated during meiosis of spermatogenesis is unknown.

We previously undertook a genetic approach to identify genes that interact with *top-2* during meiosis. A genetic suppressor screen of *top-2(it7)*-induced embryonic lethality identified 11 genetic suppressors [details of this screen are published in Bhandari *et al.* 2020 (12)]. Seven of the suppressors are point mutations in *tdpt-1*, the *C. elegans* homolog of human tyrosyl-DNA phosphodiesterase 2 (*tdp2*). Tdp2 is a multifunctional protein involved in signal transduction, transcriptional regulation, and DNA repair in mitotic cells (13, 14, 15, 16). In its role as a DNA repair enzyme, the phosphodiesterase activity of Tdp2 is involved in the removal of Top-2 trapped on DNA (1, 17, 18). Normally, Top2 activity is transient, binding to DNA very briefly. However, Top2 poisons and DNA lesions can prevent the religation activity of Top2 leading to stable Top2-DNA adducts called Top2 cleavage complexes (Top2cc) or Top2-DNA protein crosslinks (Top2-DPCs) (1, 19). In such cases, Tdp2 is required to remove the Top2-DPCs and rescue the entangled Top2 (17, 18). Unrepaired Top2-DPCs block the progression of RNA and DNA polymerases. These can lead to accumulation of recombinogenic intermediates that can catalyze the generation of deletions and translocations. Therefore, Top2-DPCs can lead to mutagenesis, neurological diseases, and carcinogenesis (20).

Human Tdp2 (41 kDa) and *C. elegans* TDPT-1 (40.9 kDa) are structurally defined by two domains: an amino-terminal ubiquitin-associated domain that binds ubiquitin or small ubiquitin-like modifier (SUMO) and a C-terminal domain that shares homology to the exonuclease-endonuclease-phosphatase nuclease superfamily (21). The C-terminal exonuclease-endonuclease-phosphatase domain is a globular folded catalytic domain, and the ubiquitin-associated domain is connected to the catalytic domain *via* a flexible linker region (17, 18). The Tdp2 phosphodiesterase mechanism occurs as a S_N_2 displacement reaction through a metal dependent hydrolysis. Tdp2 hydrolyzes 5′-tyrosyl phosphodiester linkages, cleaving off tyrosine from the 5′ end of DNA releasing the Top2 protein and freeing the DNA for subsequent repair (18, 22, 23).

Prior to our studies, *tdpt-1* had not been reported to play a role in meiosis. We previously found that TDPT-1 is expressed in the nuclei of both somatic and germ line cells of the *C. elegans* hermaphrodite (24). However, the function of TDPT-1 in spermatogenesis and its relationship to the role of TOP-2 in homologous chromosome segregation in spermatogenesis is unknown. Here, we take a genetics approach supported by biochemical analyses to determine how *tdpt-1* mutations suppress *top-2(it7)*-induced phenotypes. We found that the reduction of TDPT-1 levels [point mutations or ORF deletion] can rescue *top-2(it7)* spermatogenesis defects. TOP-2 and TDPT-1 both localize within germ cell nuclei, but do not colocalize until late meiotic prophase. TOP-2 and TDPT-1 also interact in protein complexes. Lastly, we found that the *tdpt-1* suppressing mutations impair protein stability or disrupt interactions with TOP-2 resulting in the loss of *tdpt-1* function. From these data, we propose that within the *top-2(it7)* mutant spermatogenic germline WT TDPT-1 inhibits the impaired, but not catalytically dead, TOP-2 mutant enzyme preventing the chromosome remodeling that occurs prior to the first meiotic division. The *tdpt-1* suppressing mutations allow the mutant TOP-2 protein to complete its chromosome remodeling function, rescuing the chromosome segregation defects and embryonic lethality.

## Results

### Loss of TDPT-1 function suppresses *top-2* embryonic lethality and chromosome segregation defects

**Figure 1:**
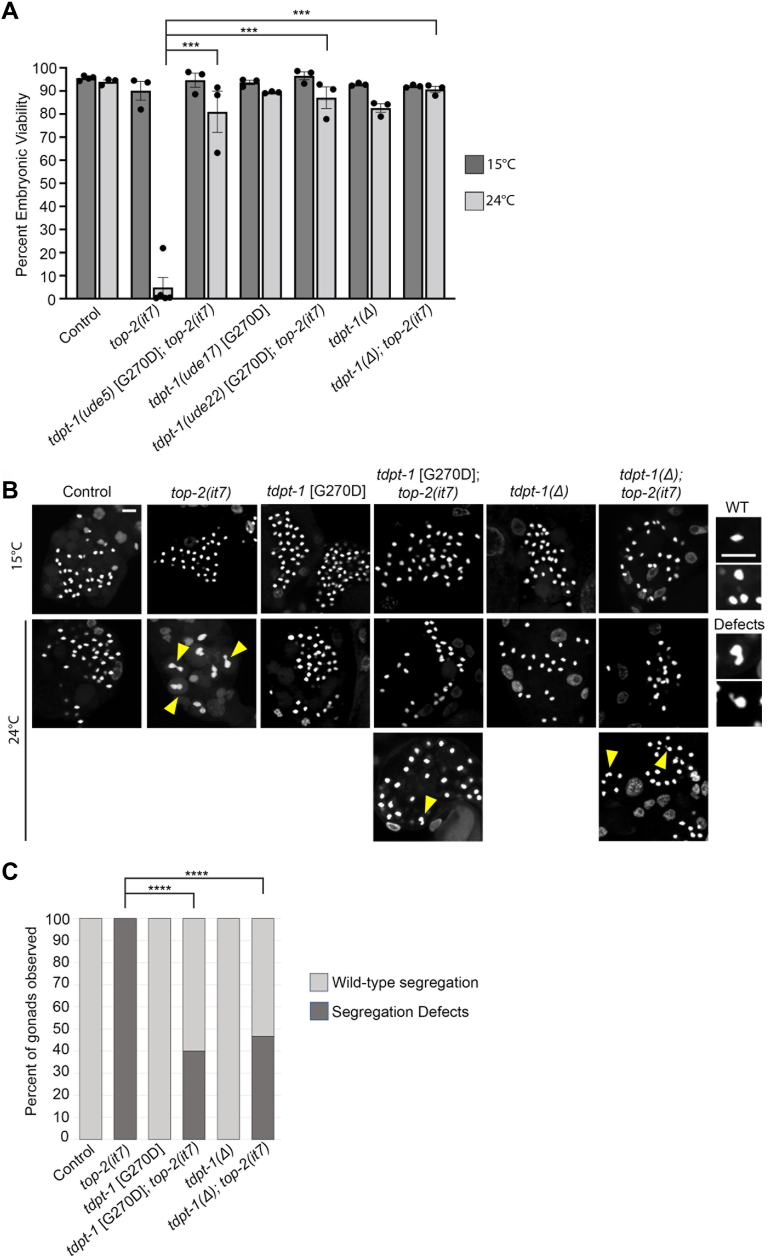
***tdpt-1* mutations alone do not cause chromosome segregation defects but can suppress *top-2(it7)* mutant phenotypes.***A*, percent embryonic viability from control [N2], *unc-4(e120) top-2(it7)*, *tdpt-1(ude5)* [G270D]; *unc-4(e120) top-2(it7), tdpt-1(ude17)* [G270D], *tdpt-1(ude22)* [G270D]; *unc-4(e120) top-2(it7), tdpt-1(tn1426Δ)*, and *tdpt-1(tn1526Δ); unc-4(e120) top-2(it7)* at 15 °C and 24 °C. The data are represented as the average of three individual replicate experiments (5–10 worms per replicate for a total of 20–30 worms per strain) with error bars representing SEM. At least 2212 progeny were scored for each genotype and temperature. *p*-Values were calculated using a Cochran-Mantel-Haenszel test with Bonferroni correction (0.00143). Any value <0.0014 is statistically significant. *B*, DNA within postmeiotic sperm of hermaphrodites 24 h post L4 at 15 °C and 24 °C. *Yellow arrowheads* indicate chromatin bridges and abnormal chromosome structures. To the *right* are enlarged examples of postmeiotic sperm with WT DNA morphology and DNA morphology indicative of segregation defects that are indicated by the *yellow arrowheads* in images to the *left*. The scale bar represents 5 μm. *C*, quantification of chromosome segregation defects at 24 °C from (*B*). Thirty germ lines were examined for each genotype. *Dark gray bars* represent the percent of germ lines with the presence of at least one chromosome segregation defect. *Light gray bars* represent the percent of germ lines with WT chromosome segregation. *p*-Values were calculated using a chi-square test comparing the *tdpt-1* mutation suppressor lines containing *top-2(it7)* with the original *top-2(it7)* line. ∗∗∗∗*p* < 0.0000001.

Previously, we identified 11 suppressors of *top-2(it7)* [R828C] embryonic lethality and confirmed that seven of those suppressors, *ude2*, *ude3*, *ude4*, *ude5*, *ude7*, *ude13*, and *ude24*, harbored single, missense mutations within the same gene, *tdpt-1* (12). In this study, we first tested if *tdpt-1* missense mutations in an otherwise WT background (in the absence of the *top-2* [R828C] mutation) cause meiotic phenotypes. The G270D mutation [*tdpt-1(ude5)* suppressor] was recreated in WT (N2) animals using CRISPR/Cas9 genome editing [new allele designation of *tdpt-1(ude17)*]. We focused on the *tdpt-1* [G270D] mutation as more than one suppressor line was identified with a mutation at this amino-acid site (*ude5*, *ude7*, and *ude24*) and it is one of the strongest suppressors (12). Embryonic viability assays and 4′,6-diamidino-2-phenylindole (DAPI) staining of postmeiotic sperm DNA showed that *tdpt-1* [G270D] did not have any obvious meiotic defects at either 15 °C or 24 °C compared to *top-2(it7)* [R828C]. The embryonic viability of *tdpt-1* [G270D] was 93.7% and 89.4% at 15 °C and 24 °C respectively *versus top-2* [R828C] (Fig. 1*A*, 90.1% and 4.9% at 15 °C and 24 °C). In addition, no meiotic chromosome segregation defects were observed in the *tdpt-1* [G270D] mutation and brood sizes were similar to the control (Figs 1, *B* and *C* and S1). To extend these results, we asked if a complete ORF deletion of *tdpt-1* [*tdpt-1(tn1526Δ)*] results in embryonic lethality or meiotic chromosome segregation defects. *tdpt-1(tn1526Δ)* had a high percentage of viable embryos at both 15 °C and 24 °C (Fig. 1*A*, 92.8% and 79.8%), brood sizes similar to control, and no chromosome segregation defects were observed in sperm (Figs. 1, *B* and *C* and S1). From these data, we conclude that *tdpt-1* mutations do not have defects in meiosis at the level of chromosome structure and segregation.

We previously determined that a reduction of *tdpt-1* through RNAi can suppress *top-2(it7)* [R828C] (referred to as R828C from here for ease of reporting the different amino-acid substitutions) embryonic lethality (12). To further validate these results, we combined *tdpt-1* mutations with the *top-2* [R828C] mutation and examined embryonic viability, brood sizes, and sperm chromosome morphology at both the permissive and restrictive temperatures. At 24 °C the embryonic viability of *top-2* [R828C] is greatly reduced compared to the control (4.9% *versus* 94.0%). However, embryonic viability significantly increases upon the introduction of the *tdpt-1* mutations [Fig. 1*A*, *tdpt-1(ude5)* [G270D]*; top-2(it7)* [R828C] *=* 81.0%, *tdpt-1(ude22)* [G270D]*; top-2(it7)* [R828C] *=* 87.1%, and *tdpt-1(tn1526Δ); top-2(it7)* [R828C] *=* 90.6%]. We also assessed postmeiotic sperm for chromosome segregation defects. While the *top-2* [R828C] worms produce sperm that display chromosome segregation defects observed in the form of chromatin bridges in 100% of the gonads observed, the introduction of *tdpt-1* mutations in the *top-2* [R828C] background resulted in a reduction in the number of gonads with chromosome segregation defects such that segregation defects were observed in less than half of the gonads observed (Fig. 1, *B* and *C*). Of note, we previously found that the original *tdpt-1* [G270D] suppressing mutation [*tdpt-1(ude5); top-2(it7)*] has less than 10% of germ lines with chromosome segregation defects (12), while, here we found that the recreated *tdpt-1* [G270D] allele [*tdpt-1(ude22); top-2(it7)*], has ∼40% of germ lines with chromosome segregation defects (Fig. 1*C*). We believe that the difference arises from the different genetic backgrounds from which these *tdpt-1* suppressing mutations were derived and from our method of quantification of chromosome segregation defects that we use. The *ude5* suppressing mutation arose from the ethyl methanesulfonate mutagenesis screen, and it is possible that there are other genetic variants within this line that modify and slightly enhance the suppression. As the *ude22* allele came from direct CRISPR/Cas9 editing of the *top-2(it7)* [R828C] strain, these may lack the additional genome variants that are present in the *ude5* strain. We also found that brood sizes for the *tdpt-1* suppressing mutants combined with *top-2* reflect that suppression is not 100% [Fig. S1, 24 °C: control = 256.4, *top-2(it7)* [R828C] = 110.5, *tdpt-1(ude5)* [G270D]*; top-2(it7)* [R828C]*=* 70.9, *tdpt-1(ude22)* [G270D]*; top-2(it7) =* 138.9]. However, the *tdpt-1* ORF deletion mutant did restore brood size to control levels [Fig. S1, 24 °C: control = 256.4 *versus tdpt-1(tn1526Δ); top-2(it7)* [R828C] *=* 265.6]. Taken together these data demonstrate that *tdpt-1* loss-of-function mutations are strong suppressors of *top-2* [R828C] embryonic lethality and chromosome segregation defects.

### TDPT-1 and TOP-2 localize to separate compartments within male germ line nuclei until late meiotic prophase

**Figure 2:**
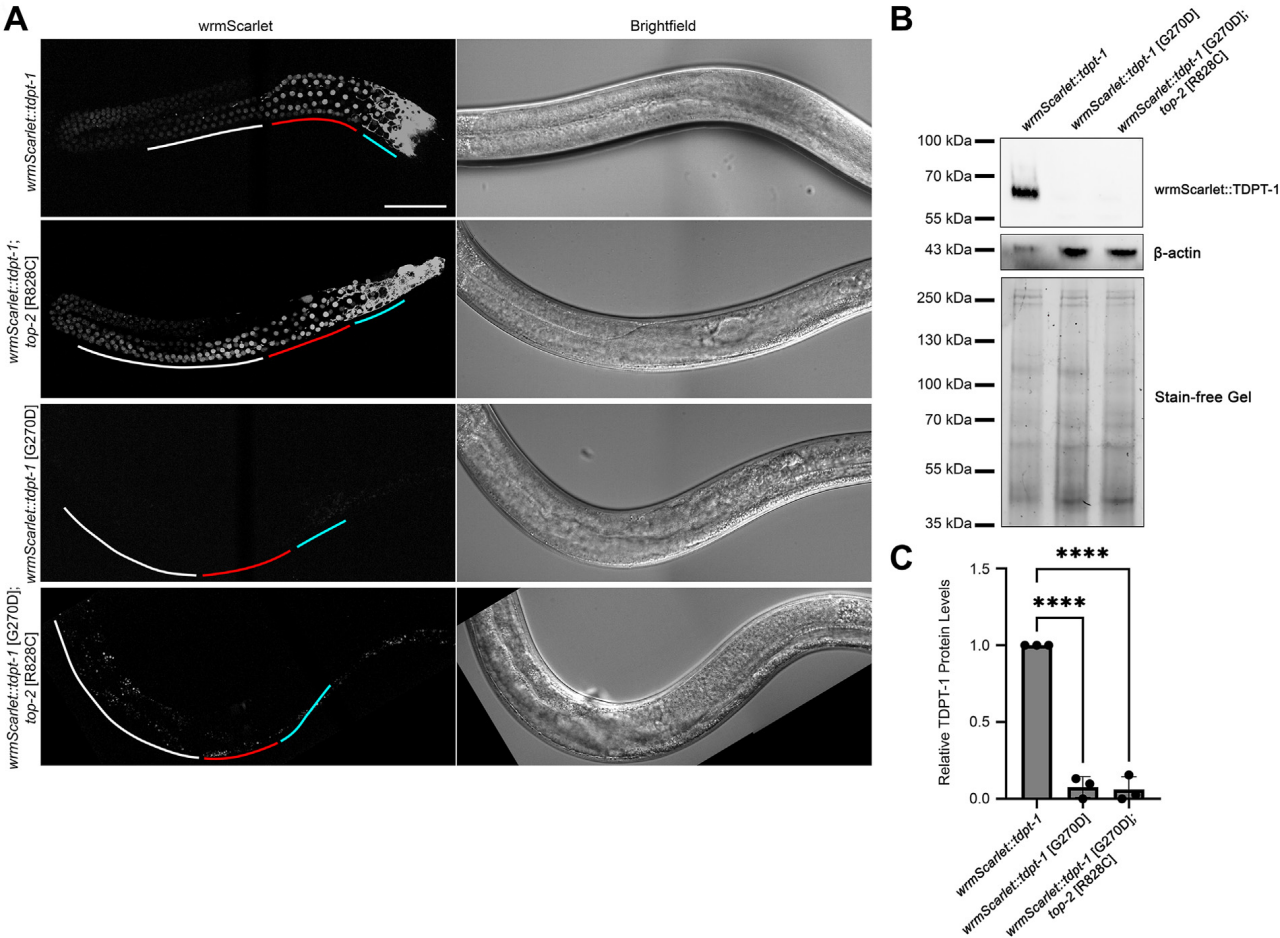
**TDPT-1 localizes to meiotic nuclei in male germ lines.***A*, localization of TDPT-1 in male germ lines visualized using spinning disc confocal imaging on endogenously tagged wrmScarlet::TDPT-1 24 h post L4 at 24 °C. Regions of the proximal gonad are labeled: pachytene (*white*), condensation zone (diplotene, karyosome, and diakinesis, *red*), and the meiotic division zone (*teal*). The scale bar represents 50 μm. Fluorescence in the *bottom* panel is from intestinal autofluorescence. *B*, Western blot of wrmScarlet::TDPT-1 (67 kDa) protein in WT and mutant animals grown at 24 °C. *C*, quantification of wrmScarlet::TDPT-1 protein levels compared to total protein (stain-free gel). Data show the average of three independent experiments. Error bars represent SEM. ∗∗∗∗*p* < 0.0001 calculated using Student’s *t* test.

We previously generated a line with a fluorescently tagged TDPT-1 protein (wrmScarlet::TDPT-1) and found that TDPT-1 is expressed in both somatic and germ line nuclei of *C. elegans* hermaphrodites (24). To determine the localization of TDPT-1 in the male germ line, we visualized wrmScarlet::TDPT-1 relative to brightfield images of intact males. TDPT-1 localized to the nucleoplasm of both somatic and germ line nuclei (Fig. 2*A*). Since previous reports found that in somatic cells TDPT-1 removes TOP-2-DPC from DNA, we asked if the localization of TDPT-1 changes in spermatogenesis in the *top-2* loss-of-function mutant. In *top-2* [R828C] mutant germ lines, TDPT-1 localization did not change and remained nucleoplasmic (Fig. 2*A*). We also used CRISPR/Cas9 genome editing to recreate the *tdpt-1* [G270D] mutation in the wrmScarlet::TDPT-1 line. The mutant wrmScarlet::TDPT-1 [G270D] protein was not detected (Fig. 2*A*). To determine if the TDPT-1 [G270D] mutation affects protein levels, we performed a Western blot on whole worm lysates from *wrmScarlet::tdpt-1*, *wrmScarlet::tdpt-1* [G270D], and *wrmScarlet::tdpt-1* [G270D]*; top-2* [R828C]. Using an anti-red fluorescent protein (RFP) antibody, wrmScarlet::TDPT-1 protein was only detected in *wrmScarlet::tdpt-1* (Fig. 2, *B* and *C*). TDPT-1 protein levels were greatly reduced in both *wrmScarlet::tdpt-1* [G270D] and *wrmScarlet::tdpt-1* [G270D]*; top-2* [R828C] (Fig. 2, *B* and *C*). These data further confirm that knockdown of TDPT-1 (*i.e.*, protein levels as seen in the G270D mutation or by the null mutant) is one mechanism that mediates suppression of *top-2* [R828C] spermatogenesis defects.

**Figure 3:**
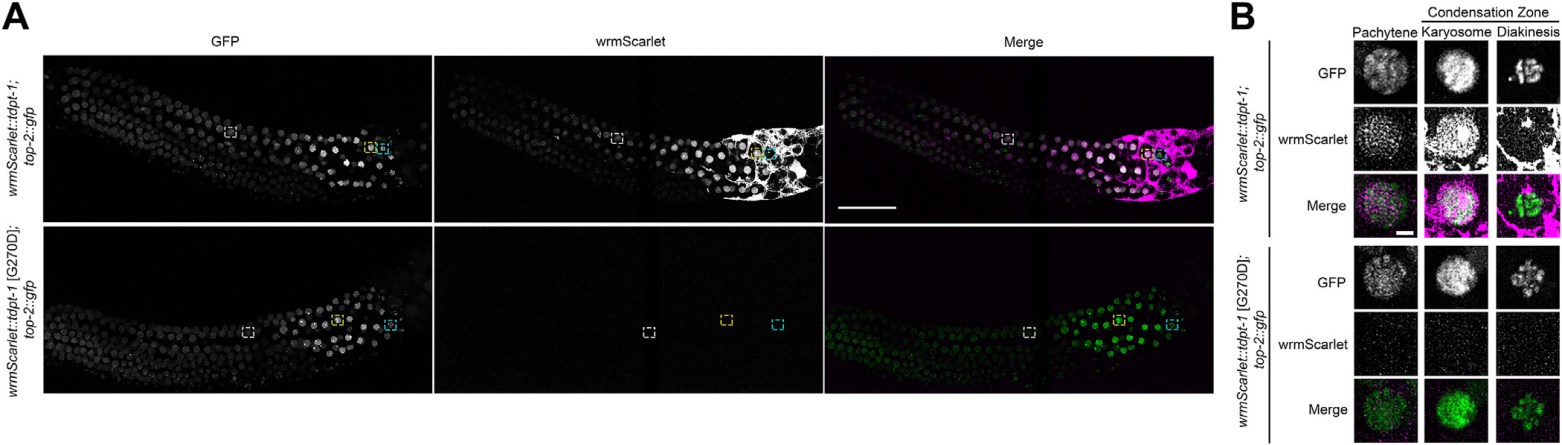
**TDPT-1 and TOP-2 do not colocalize in the nuclei of male germ lines.** Localization of TDPT-1 and TOP-2 visualized using spinning disk confocal imaging on endogenously tagged wrmScarlet::TDPT-1 (*magenta*) and TOP-2::GFP (*green*). *A*, localization of wrmScarlet::TDPT-1 and TOP-2::GFP within meiotic nuclei in the male gonad. The scale bar represents 50 μm. *B*, enlarged images of germ line nuclei in pachytene (*white box*) and in the condensation zone (karyosome= *yellow box*, diakinesis= *cyan box*). The scale bar represents 2.5 μm. *wrmScarlet::tdpt-1(+); top-2(+)::gfp*: N = 39 and *wrmScarlet::tdpt-1* [G270D]; *top-2(+)::gfp*: N = 39. N is the number of male germ lines examined for each genotype.

Previously, we determined the localization pattern of TOP-2 in male germ lines. In control males, TOP-2 localizes along chromosomes in prophase, however the *top-2* [R828C] mutation disrupts the association of TOP-2 with chromosomes in spermatogenesis (3, 25). As germline nuclei transition from mitosis in the proliferative zone to meiosis in *top-2* [R828C] germ lines, TOP-2 fails to associate with chromosomes and remains nucleoplasmic (3). To determine if the *tdpt-1* suppressing mutations can restore TOP-2 localization to meiotic chromosomes, we visualized TOP-2::GFP during spermatogenesis in animals expressing wrmScarlet::TDPT-1 and wrmScarlet::TDPT-1 [G270D]. In the genetic backgrounds where both proteins harbor WT amino-acid sequences, TOP-2 localizes along chromosomes while TDPT-1 is nucleoplasmic from the distal tip through midmeiotic prophase I (Fig. 3). In many prophase nuclei a single TOP-2::GFP focus is present, which was also previously observed in meiotic prophase germ cells of the hermaphrodite germline (25). The source of these foci is unknown; however, these foci are not present in germ lines immunostained for TOP-2 localization using an anti-FLAG antibody in TOP-2::3XFLAG tagged lines (3). Next, we examined TOP-2::GFP (WT amino-acid sequence) localization in the presence of TDPT-1 carrying a *top-2* suppressing mutation (wrmScarlet::TDPT-1 [G270D]). TOP-2::GFP localization was unchanged; localizing to chromosomes in meiotic prophase, however, as observed in Figure 2, TDPT-1 protein was not detected (Fig. 3).

**Figure 4:**
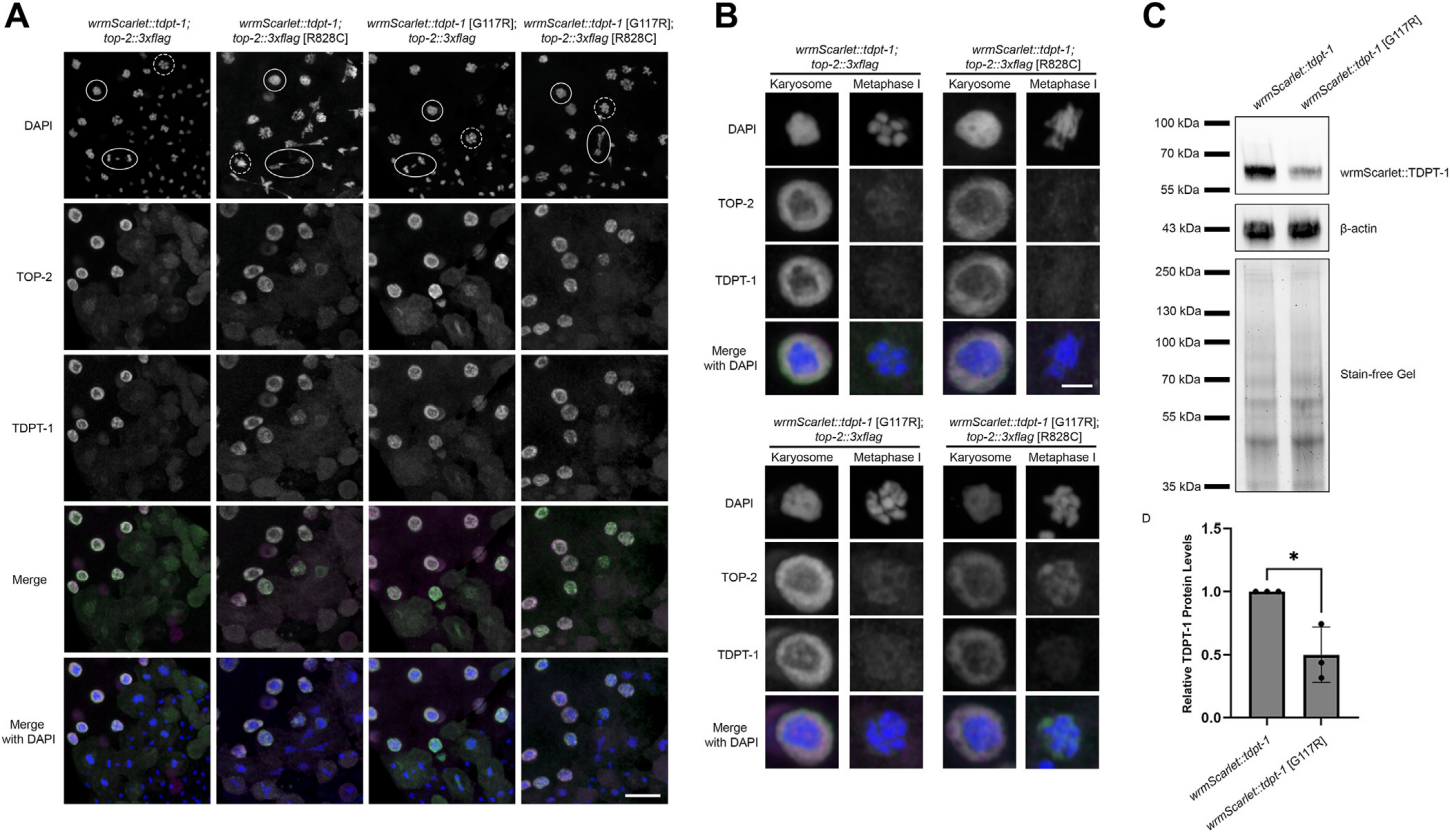
**TDPT-1 [G117R] localizes to the nucleoplasm of male germ cells at reduced levels.***A*, immunostaining of TOP-2::3XFLAG (*green*) and wrmScarlet::TDPT-1 (*magenta*) counterstained with DAPI *(blue*). Half z-stack projections of control (*wrmScarlet::tdpt-1; top-2::3Xflag*) *versus wrmScarlet::tdpt-1; top-2* [R828C]*::3Xflag*, *wrmScarlet::tdpt-1* [G117R]*; top-2::3Xflag*, and *wrmScarlet::tdpt-1* [G117R]*; top-2* [R828C]*::3Xflag* in dissected male germlines. The scale bar represents 10 μm. *Solid circles* represent karyosome nuclei, *dashed circles* represent metaphase I nuclei, and *ovals* represent anaphase I nuclei. Sample numbers for *wrmScarlet::tdpt-1; top-2::3Xflag*: N = 17, *wrmScarlet::tdpt-1; top-2 [R828C]::3Xflag*: N = 15, *wrmScarlet::tdpt-1* [G117R]*; top-2::3Xflag*: N = 20, and *wrmScarlet::tdpt-1* [G117R]*; top-2* [R828C]*::3Xflag*: N = 17. *B*, enlarged images of encircled karyosome (*solid circle*) and metaphase I (*dashed circles*) germ line nuclei from (*A*). The scale bar represents 2 μm. *C*, Western blot of wrmScarlet::TDPT-1 (67 kDa) protein levels in control (wrmScarlet::TDPT-1; TOP-2::3XFLAG) and *tdpt-1* mutant animals (wrmScarlet::TDPT-1 [G117R]; TOP-2::3XFLAG) grown at 24 °C. *D*, quantification of wrmScarlet::TDPT-1 protein levels compared to total protein (stain-free gel). Data show the average of three independent experiments. Error bars represent SEM. ∗*p* < 0.05 calculated using Student’s *t* test. DAPI, 4′,6-diamidino-2-phenylindole.

Next, we wanted to determine the localization pattern of the mutant TOP-2 protein (TOP-2 [R828C]::GFP) in relation to the localization of control (wrmScarlet::TDPT-1) and mutant TDPT-1 (wrmScarlet::TDPT-1 [G270D]). However, we were unable to recover viable homozygous lines of the mutant version of the tagged TOP-2 protein. We performed whole mount DAPI staining of homozygous *top-2* [R828C]*::gfp* hermaphrodites recovered from *top-2* [R828C]*::gfp/mnC1* mothers. Imaging revealed that these animals have severely disrupted germ lines (Fig. S2*A*). Germline defects lead to the production of low numbers of embryos and the few embryos that are produced are not viable (Fig. S2, *B* and *C*). While TOP-2::GFP animals are viable and fertile (25), the addition of the R828C missense mutation along with the GFP tag impairs the function of TOP-2 even at permissive temperatures (15–20 °C). Therefore, we turned to immunostaining to determine the localization relationship of TOP-2 and TDPT-1 utilizing a 3XFLAG tagged TOP-2 [R828C] that we previously demonstrated is viable and fertile (3). Similar to the live imaging experiments, immunostaining (using anti-RFP and anti-FLAG antibodies) of wrmScarlet::TDPT-1; TOP-2::3XFLAG animals revealed that TDPT-1 is nucleoplasmic throughout the germ line while TOP-2 localizes along chromosomes throughout meiotic prophase during spermatogenesis. In late meiotic prophase (karyosome stage), both TDPT-1 and TOP-2 are nucleoplasmic, with a concentrated ring surrounding the karyosome DNA (Fig. 4). We also examined the localization of another *tdpt-1* suppressor mutation, *tdpt-1(ude4)* [G117R] in relation to TOP-2 localization. TDPT-1 [G117R] is expressed and nucleoplasmic in the presence of TOP-2::3XFLAG (Fig. 4). In wrmScarlet::TDPT [G117R]; TOP-2 [R828C]::3XFLAG male germ lines, there was no change in the localization of the mutant TOP-2 protein; however, chromosome structure was mostly restored with compact spermatids present (Fig. 4). These results were also reflected in the rescue of *top-2(it7)*-induced embryonic lethality. Embryonic viability was low in *wrmScarlet::tdpt-1(+)*; *top-2(it7)* [R828C] (Fig. S3). However, suppression of embryonic lethality was observed in *wrmScarlet::tdpt-1* [G117R]; *top-2(it7)* [R828C] (Fig. S3). In addition, a Western blot was performed to determine if the G117R mutation affected TDPT-1 protein levels. TDPT-1 protein levels were reduced in *wrmScarlet::tdpt-1* [G117R] (Fig. 4, *B* and *C*).

### Most *tdpt-1* mutations impair the phosphodiesterase catalytic activity of TDPT-1

**Figure 5:**
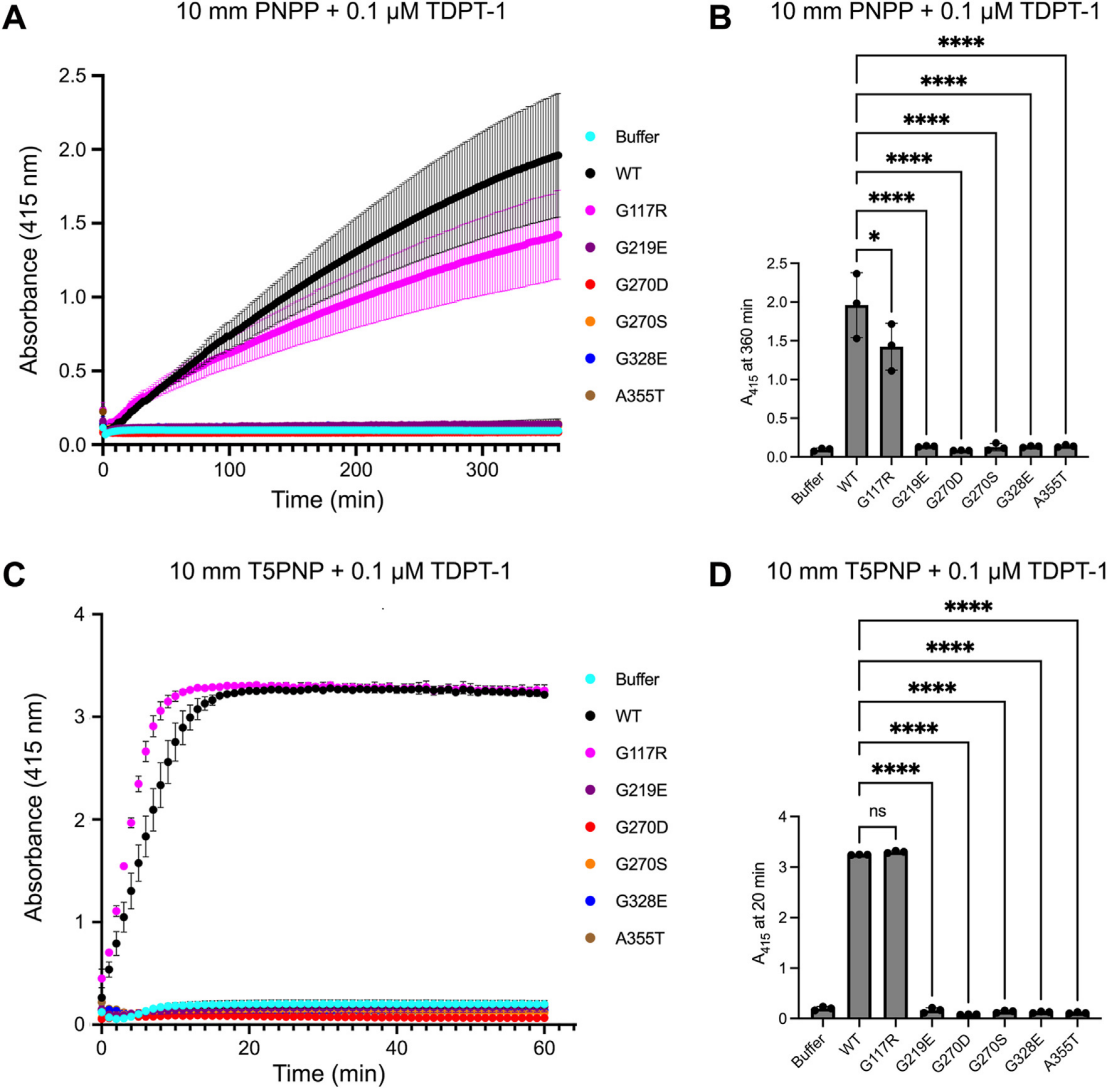
**Most *tdpt-1* suppressing mutations impair phosphodiesterase catalytic activity.***A*–*D*, phosphodiesterase activity of TDPT-1 on the chemical substrates PNPP (para-nitrophenyl phosphate) and T5PNP (thymidine 5′-monophosphate p-nitrophenyl ester), yields PNP (para-nitrophenol), which gives off a *yellow color*. This was measured as an increase in absorbance at 415 nm over time for TDPT-1 proteins with PNPP (*A* and *B*) and T5PNP (*C* and *D*) substrates. The chromogenic phosphodiesterase assay was performed in triplicate for each protein. PNPP, p-nitrophenyl phosphate; T5PNP, thymidine 5′-monophosphate p-nitrophenyl ester.

Human Tdp2 and *C. elegans* TDPT-1 both have 362 amino acids. Human Tdp2 specifically cleaves tyrosine from the 5′- end of DNA protein cross links, leaving a phosphate group available for religation thus resolving 5′-phosphotyrosyl covalent adducts (23). Notably, all of the *tdpt-1* suppressing mutations are found within the approximately 250 amino-acid residues that make up the catalytic domain. To evaluate the impact of the *tdpt-1* mutations on the phosphodiesterase activity of the TDPT-1 protein, *C. elegans* WT and mutant TDPT-1 proteins were codon optimized and expressed in an *Escherichia coli* system (Fig. S4) (17). Purified TDPT-1 protein was used in an *in vitro* chromogenic 5′-phosphotyrosine hydrolysis assay using two substrates, p-nitrophenyl phosphate (PNPP) and thymidine 5′-monophosphate p-nitrophenyl ester (T5PNP) (22). This *in vitro* phosphodiesterase activity assay showed that five out of the six *tdpt-1* mutations (G219E, G270D, G270S, G328E, and A355T) have impaired catalysis on both chemical substrates with flat curves corresponding to no absorbance (Fig. 5, *A* and *C*). Only the G117R mutation exhibited TDPT-1 phosphodiesterase activity with comparable activity to WT TDPT-1 (Fig. 5, *A*–*D*). These data suggest that five of the identified mutations (G219E, G270D, G270S, G328E, and A355T) are catalytically dead proteins.

### *Tdpt-1* suppressing mutations affect the thermal stability of TDPT-1

**Figure 6:**
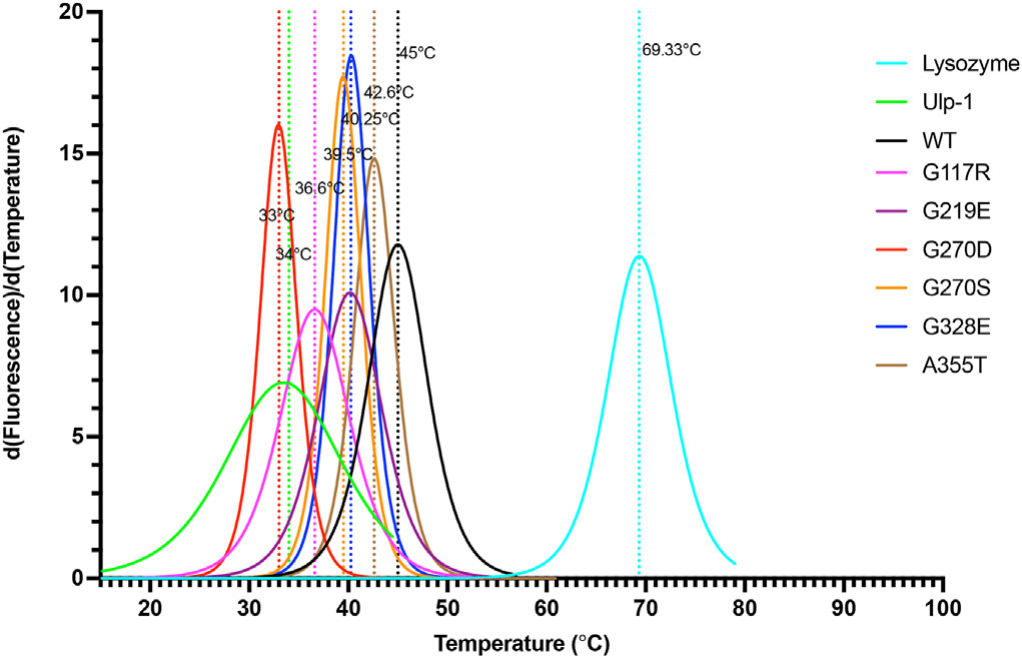
***tdpt-1* suppressing mutations affect the thermal stability of TDPT-1 protein.** The first derivative of the nonlinear fit of normalized melting fluorescence data was obtained to evaluate the Tm of the TDPT-1 proteins. Lysozyme and Ulp-1 were used as controls. Different mutations in TDP-1 protein and the corresponding Tm are shown. Three replicates were used for each protein.

Amino-acid substitutions of even a single amino-acid can have a significant impact on the folding, aggregation, and stability of proteins, which can be either favorable or unfavorable (26, 27). To assess the impact of the *tdpt-1* point mutations on the thermal stability of TDPT-1, a thermal shift assay was performed to determine the Tm of mutants relative to the WT TDPT-1 protein. While a thermal shift assay is used primarily to optimize buffers and identify ligands, here, the thermal denaturation temperature was measured while keeping the other conditions constant (*e.g.*, pH and buffers). The thermal stability of proteins is displayed as the Tm, which corresponds to the temperature where the protein is 50% unfolded. WT TDPT-1 possessed a higher thermal stability than the mutant TDPT-1 mutations (Fig. 6). The control proteins lysozyme and Ulp-1 have Tm at 69.3 °C and 34 °C, respectively. The Tm of WT TDPT-1 is 45 °C while all of the mutant TDPT-1 proteins have lower Tm values (Fig. 6). Among the mutant TDPT-1 proteins, the G270D mutant has the lowest Tm at 33 °C while the A355T mutant has the highest Tm at 42.6 °C. Both G219E and G328S have a Tm of 40.25 °C. Interestingly, the G117R mutant, which is the only mutant that retains phosphodiesterase activity has a Tm lower than all of the mutant TDPT-1 proteins except G270D (Fig. 6). This thermal instability is reflected in the lower protein levels observed in Figure 4.

### TDPT-1 [G117R] disrupts TOP-2-TDPT-1 protein complexes

**Figure 7:**
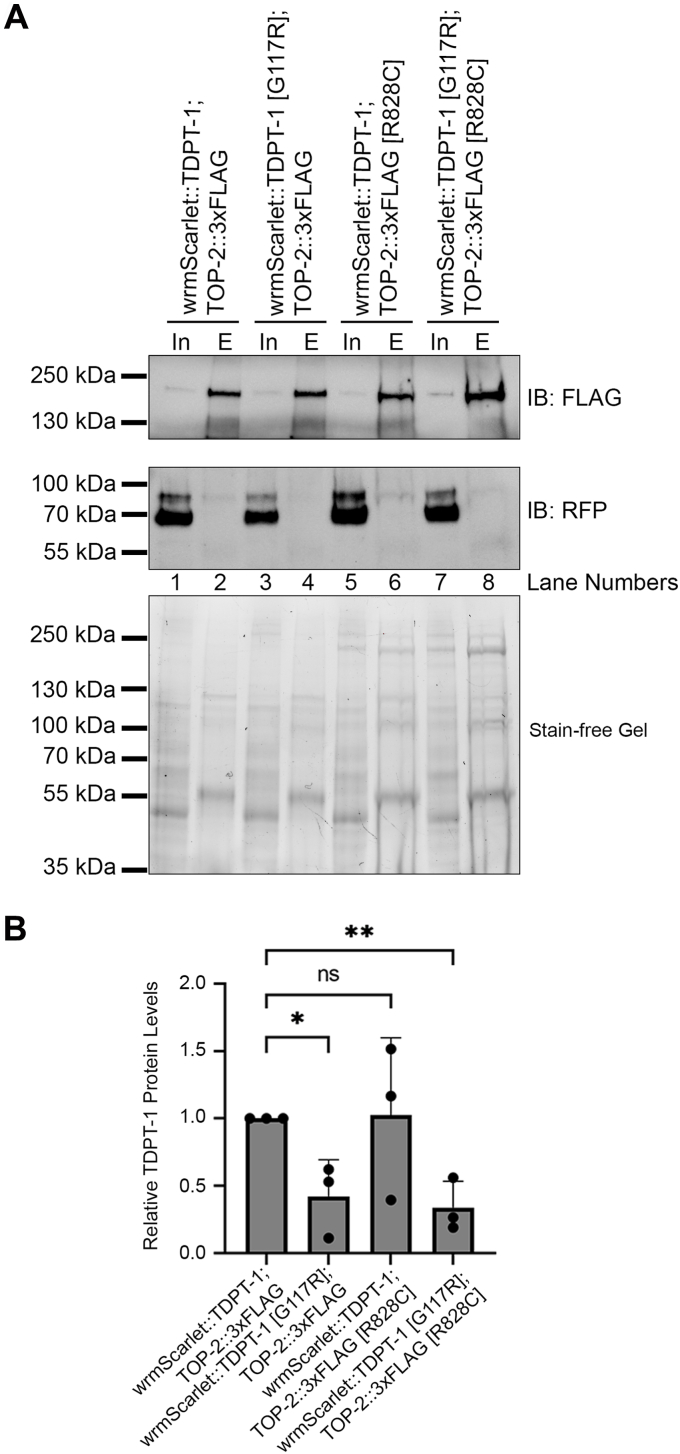
**TDPT-1 [G117R] disrupts interactions with TOP-2.***A*, Coimmunoprecipitation and immunoblotting (IB) of wrmScarlet::TDPT-1 and TOP-2::3xFLAG using anti-FLAG magnetic agarose beads. The anti-FLAG immunoblot shows TOP-2::3xFLAG and TOP-2 [R828C]::3xFLAG (180 kDa) in both the input (In) and eluate (E). The anti-RFP immunoblot detects wrmScarlet::TDPT-1 and wrmScarlet::TDPT-1 [G117R] (67 kDa) in the input. The wrmScarlet::TDPT-1 protein pulled down in TOP-2 IPs is of a higher molecular weight than the predicted wrmScarlet::TDPT-1 (∼85 kDa). *B*, quantification of wrmScarlet::TDPT-1 protein levels in the eluate compared to total protein levels (stain-free gel). ∗*p* < 0.05, ∗∗*p* < 0.005. P- Values were calculated using Student’s *t* test. IPs, immunoprecipitants; REP, red fluorescent protein.

While the *tdpt-1* [G117R] mutant has a lower thermal stability and lower protein levels than control *tdpt-1* [*wrmScarlet::tdpt-1(+)*], it is expressed and has a similar localization pattern as the control (Figs. 4 and 6). TDPT-1 [G117R] is also the only mutant that retains phosphodiesterase activity (Fig. 5). We next asked whether the G117R mutation disrupts TOP-2-TDPT-1 protein-protein interactions. In order to confirm a physical interaction between TDPT-1 and TOP-2, we conducted co-immunoprecipitation (co-IP) experiments and detected wrmScarlet::TDPT-1 in complexes isolated from TOP-2::3xFLAG immunoprecipitants (IPs) (Fig. 7). An interaction was detected in the context of both TDPT-1 and TOP-2 control proteins and in the context of the mutant TOP-2 [R828C] protein (lanes 2 and 6). The reciprocal interaction was also observed in IPs of wrmScarlet::TDPT-1 (Fig. S5*A*). Surprisingly, the wrmScarlet::TDPT-1 pulled down with TOP-2 complexes is a higher molecular weight band than the predicted wrmScarlet::TDPT-1 (Fig. 7, lane 2). The higher molecular weight band is also observed in the input (Fig. 7, lane 1). This suggests that TDPT-1 may be modified prior to interacting with TOP-2. The modified TDPT-1 does not appear to be due to SUMOylation or ubiquitination (Fig. S5, *B* and *C*). Additional studies will need to be done in the future to determine the TDPT-1 posttranslational modification. To test if the *tdpt-1* [G117R] mutation disrupts TDPT-1-TOP2 protein complexes we performed co-IPs in *wrmScarlet::tdpt-1* [G117R] strains. A reduced amount of wrmScarlet::TDPT-1 [G117R] was detected in TOP-2::3XFLAG IPs (Fig. 7, lanes 4 and 8). These results suggest that TOP-2 and TDPT-1 physically interact in *C. elegans* and that the TDPT-1 [G117R] suppressing mutation disrupts this interaction.

## Discussion

We previously reported the identification of seven *tdpt-1* mutations that suppress *top-2(it7)* [R828C]-phenotypes. In this study, we explored the mechanism of suppression of *tdpt-1* mutations on *top-2* [R828C] chromosome segregation defects and embryonic lethality. Using a combination of genetic and biochemical approaches we found that the loss of *tdpt-1* function suppresses the *top-2* [R828C] defects. Recreation of the *tdpt-1* suppressing mutation G270D in an otherwise WT genetic background does not cause meiotic defects or embryonic lethality. Furthermore, a *tdpt-1* ORF deletion had no impact on meiosis or embryonic lethality. However, when these same *tdpt-1* mutations are introduced into the *top-2* [R828C] genetic background, chromosome segregation defects are ameliorated, and embryonic viability of the progeny is rescued (Fig. 1).

**Figure 8:**
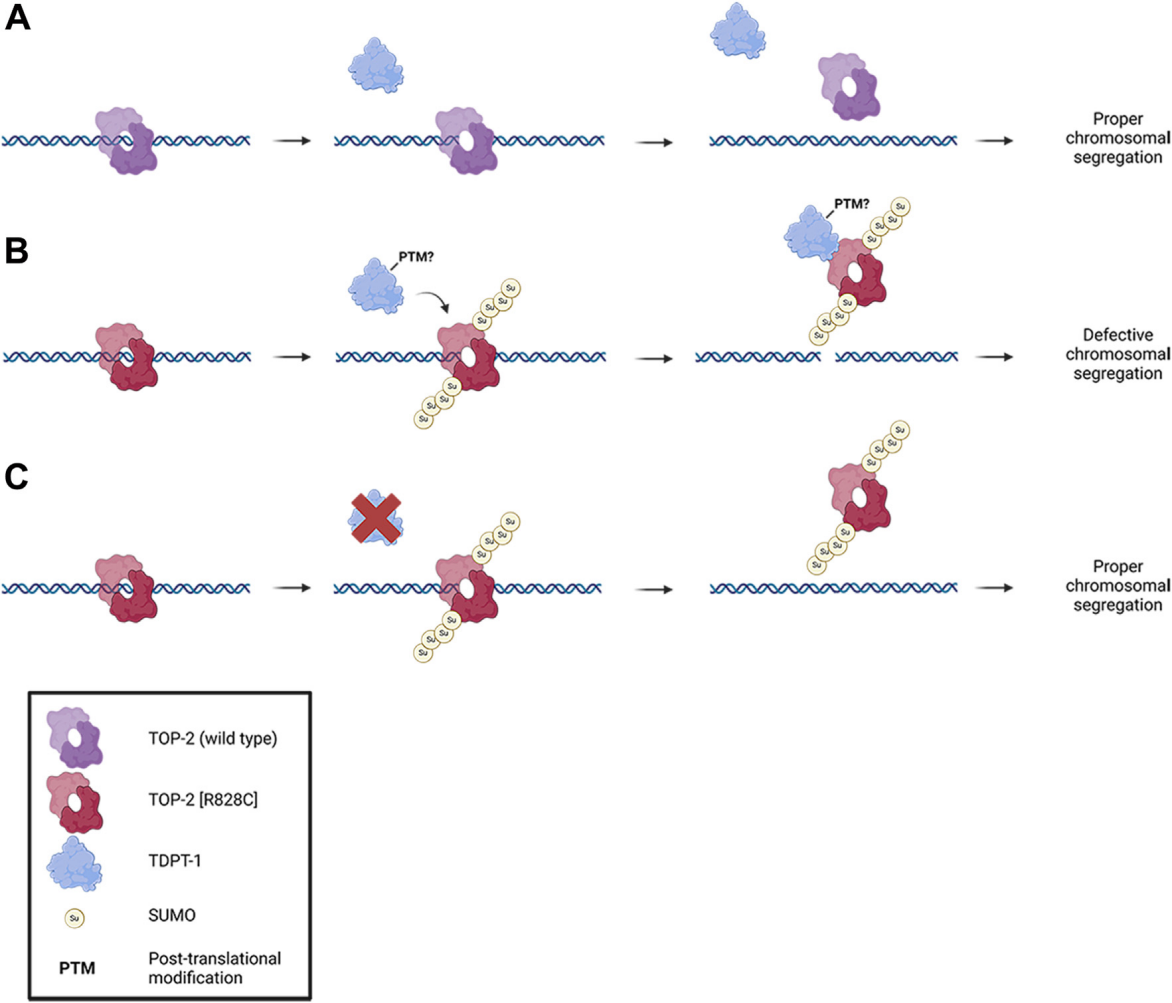
**Model for TOP-2 and TDPT-1 interplay during spermatogenesis.***A*, WT TOP-2 homodimers (*purple*) bind to DNA to introduce transient DSBs required for adequate chromosome remodeling that ensures proper chromosome segregation. TDPT-1 (*blue*) is not required under this condition. *B*, the TOP-2 [R828C] mutation makes a defective protein (*red*) that is slow to complete its function; therefore, it appears trapped with DNA (TOP-2-DPC). Potentially, there are specific modifications (*e.g.*, SUMOylation) of TOP-2 that allow for the recruitment of TDPT-1 and other proteins, such as adaptors or chaperones that induce the release of TOP-2 from the DNA. WT TDPT-1 quickly resolves these TOP-2 DPCs. Early release of TOP-2 prevents any residual activity of TOP-2 [R828C] from occurring, ultimately leading to a failure in chromosome segregation. *C*, when TDPT-1 is absent or inactive (red X) due to mutations, this allows for the residual activity of TOP-2 to complete its functions and ameliorates the chromosome segregation defects. DSB, double-strand break; DPC, DNA protein crosslink; SUMO, small ubiquitin-like modifier.

Why is it that the loss of TDPT-1 alone does not affect meiosis and only produces a phenotype in relation to loss of *top-2* function? From previous studies, the main function of TDPT-1 is to remove TOP-2-DPCs. When meiosis is proceeding under normal circumstances, TDPT-1 is not required to remove the TOP-2-DPCs as these are transient. TOP-2 is able to perform its chromosome remodeling function in late prophase I, which ensures accurate chromosome segregation. Therefore, the loss of TDPT-1 when TOP-2 is functioning properly does not affect chromosome segregation. In the case of the *top-2(it7)* [R828C] mutant, we previously demonstrated that TOP-2 protein levels are reduced (12) and the remaining TOP-2 [R828C] protein is not detected on the chromosomes of meiotic prophase I as it is in WT animals (3). While localization is not detected at the level of immunostaining and confocal microscopy, we do not know if any of the remaining residual amounts of TOP-2 bind to DNA. Nevertheless, this study and our previous work suggest that the R828C mutation causes a reduction in function of the TOP-2 protein rather than a complete absence of function [*e.g.*, Fig. 4, (3, 12)]. We hypothesize that the R828C amino-acid substitution, not only disrupts protein localization, but may affect TOP-2 enzymatic kinetics resulting in a slower functioning TOP-2. In this scenario we propose that some TOP-2 [R828C] protein may still bind DNA and introduce DSBs but may then fail to passage the other DNA strand and religate the DSBs in a timely manner, thereby making the transient TOP2-DPCs look like permanent TOP2-DPCs (Fig. 8). TDPT-1 recognizes these TOP2-DPCs, but in the process of removing the DPCs, it abrogates the residual activity of TOP-2 [R828C], resulting in chromosome segregation defects and very low embryonic viability (Fig. 8).

How do *tdpt-1* mutations suppress *top-2(it7)* [R828C] defects? We propose that the absence of TDPT-1 activity creates an environment conducive for the slower TOP-2 [R828C] to perform its roles in meiosis (Fig. 8). Consistent with this, both *tdpt-1*(*tn1526Δ)* and *tdpt-1* [G270D], which have a complete absence of TDPT-1, as well as the *tdpt-1* [G117R], with reduced TDPT-1 levels and abrogated TOP-2 interactions, increased embryonic viability and ameliorated chromosome segregation defects in the *top-2(it7)* background (Figs. 1, 4, and S3). We also performed biochemical assays that support the model that loss of TDPT-1 is the mechanism that allows for *top-2* [R828C] suppression. All *tdpt-1* suppressing mutations, except for G117R, rendered TDPT-1 catalytically dead (Fig. 5). Additionally, thermal shift assays showed that the *tdpt-1* suppressing mutations affect the stability of TDPT-1. The Tm values of all the TDPT-1 mutant proteins were less than the WT TDPT-1 (Fig. 6). Interestingly, G117R was the only mutant protein that exhibited phosphodiesterase activity. However, the Tm value of G117R was lower than most of the mutant TDPT-1 proteins, only G270D had a lower Tm, and had reduced protein levels. This indicates that G117R may not be required for phosphodiesterase activity but is required for the stability of TDPT-1 protein. Additionally, we found that the G117R mutation disrupts TOP-2-TDPT-1 interactions further supporting our model that preventing TDPT-1 interactions with the TOP-2 [R828C] mutant (*i.e.*, lack of expression or blocking protein interactions) suppresses *top-2* [R828C] phenotypes.

Another question that remains is how TDPT-1 recognizes TOP-2-DPCs. A previous study found that TOP2 must be partially proteolyzed before TDP2 can access the DNA-5′-phosphotyrosyl bond and TDP2 is predicted to have an ubiquitin-like domain at its N-terminus (23, 28). However, a more recent study found that TDP2 interacts with a protein called ZATT/ZNF451, a SUMO E3/E4 ligase/elongase (29). In this case, ZATT/ZNF451 may be acting as a chaperone through the SUMOylation of TOP2-DPCs, thereby changing the conformation of the TOP2-DPCs that allows TDP2 access to the phosphotyrosyl bond (29). At this time, a ZATT/ZNF451 homolog has not been identified in *C. elegans*. Whether a similar mechanism exists in the worm germ line is not known. Of note, we found that TOP-2 interacts with a modified TDPT-1 protein (Fig. 7). Future studies will determine the identity of the posttranslational modification and if this is important for TDPT-1-TOP-2 interaction. In addition, whether *C. elegans* TOP-2 is SUMOylated or ubiquitinated is not known. Further studies looking at *C. elegans* TOP-2 posttranslational modifications will help clarify its role and regulation during meiosis. It has also been postulated that both SUMO and ubiquitinated forms of TOP2 may exist, and the employment of a specific modification depends on the cell cycle (30). This was proposed at the time because SUMOylation of TOP2 had been demonstrated in mitotic cells (31). More recently, TOP2-SUMOylation has been demonstrated in meiosis (32, 33) suggesting that TOP2 SUMOylation *versus* ubiquitination and the resolution of TOP-2-DPCs is more complex than the cell cycle being utilized.

Another consideration is TOP2 and repair pathway choice. TOP2-DPCs can be repaired either through nonhomologous end joining mediated by Ku and ligase IV after TDP2 removal of TOP2 or by homologous recombination (HR) through an endonuclease pathway that removes a segment of DNA with TOP2 covalently attached to it [reviewed in (34)]. Proposed models for repair pathway choice have again focused on the cell cycle (mitosis *versus* meiosis) and cell cycle stage (G1/S *versus* G2/M). In these models, the absence of a sister chromatid (G1/S) or close homolog when TOP2-DPCs are present relies on another DPC proteolyzing enzyme, Spartan, to remove TOP2-DPCs (35, 36). In G2/M when chromosomes have been replicated, SUMOylation of TOP2-DPCs utilize TDP2 for removal and nonhomologous end joining for repair (36). In the germline, both sister chromatids and homologous chromosomes are present, and error-free repair pathways such as HR are preferred. Recent studies have proposed that in the germline TDP2 is suppressed (36, 37). Germ cell nuclear acidic peptidase (GCNA) was found to affect accumulation of DPCs (36, 37) that include TOP2 (36). In mice, it was proposed that GCNA which lacks the Spartan domain, binds MRE11 to process TOP-2-DPCs (38). Interestingly, our data does not support the idea of TDP2 suppression in the *C. elegans* germ line and may be present as a fail-safe mechanism if HR components are not available (*i.e.*, mutations). MRE-11 as part of the Mre11-Rad50-Nbs1 complex is found in the germ line and is required for meiotic processes (39, 40), however, whether it interacts with TOP-2 during meiosis is yet to be discovered. Additional studies, to determine the interaction of TOP-2, TDPT-1 (TDP2), GCNA, and the Mre11-Rad50-Nbs1 complex in the germline will help determine how DPCs are resolved to prevent chromosome missegregation and aneuploidy in gametes.

## Experimental procedures

### Strains

*C. elegans* strains (Table S1) were maintained using standard culturing conditions (41) on Modified Youngren’s, Only Bacto-peptone (MYOB) media.

### Embryonic viability assays

As described in Kwah and Jaramillo-Lambert, 2023 (42) individual L4 hermaphrodites were placed onto 35 mm MYOB plates that were spotted with OP50. Each hermaphrodite was incubated at 15 °C or 24 °C for 24 h. The hermaphrodites were transferred to a new plate after 24 h, and this step was repeated until no additional embryos were produced. Percent viable progeny was calculated by dividing the number of hatched embryos by the total number of embryos laid.

### CRISPR methods

CRISPR-mediated genome editing was completed *via* the clone-free method (43) using *dpy-10* as a coinjection marker. Injections were completed using a mix of Cas9 protein (2.5 μg, Integrated DNA Technologies), *dpy-10* CRISPR RNA (crRNA) (25 μM, Integrated DNA Technologies), *dpy-10(cn64)* repair oligonucleotide (22 μM), universal tracrRNA (60 μM, Integrated DNA Technologies), an allele-specific crRNA (50 μM) and an allele-specific repair oligonucleotide (28 μM). See Table S2 for specific crRNA and repair templates used in the individual CRISPR mutants.

### Spinning disk confocal live imaging

Live imaging was conducted using the Andor Dragonfly Spinning Disk and Super Resolution Microscope. Male worms were prepared by chemical immobilization on a 2% agarose pad with 20 mM tetramisole (Sigma-Aldrich, T1512). After immobilization, the proper focal plane was chosen and imaged at a Z-stack interval of 0.5 μm. Image processing and analysis was conducted using Imaris Microscope Image Analysis software (Oxford Instruments, https://imaris.oxinst.com/) and Fiji (https://imagej.net/software/fiji/) (44). All images were obtained under identical conditions and parameters, with brightness and contrast adjusted to allow for better visualization.

### Immunostaining

Male germline dissection, immunofluorescence, and DAPI staining was executed as described in Rourke and Jaramillo-Lambert, 2022 (45). However, incubations at the nonpermissive temperature of 24 °C were done for 16-24 h instead of 4 h. Gonads were dissected in 30 μl of egg buffer and 0.1% Tween 20 on a glass coverslip and 15 μl of the buffer was removed postdissection and replaced with 2% paraformaldehyde. Subsequently, 15 μl of the buffer was removed, and a slide was placed on top of the coverslip and the samples allowed to fix for 5 min. Slides were then immersed in liquid nitrogen. Frozen coverslips were quickly removed and slides were placed in −20 °C 100% methanol for 1 minute. Slides were then washed once in 1× PBS + 0.5% Triton X-100 for 10 min, and then washed twice in 1× PBS + 0.1% Tween 20 (PBST) for 5 min. The slides were blocked in 0.7% bovine serum albumin in PBST for 1 hour. After blocking, 50 μl of primary antibody [mouse anti-FLAG (1:500, Sigma-Aldrich, F1804) and rabbit anti-RFP (1:100, Invitrogen, PA5-34974)] was added. A parafilm coverslip was placed on top of the samples and the slides were incubated overnight in a dark humid chamber. After the overnight primary antibody incubation, the slides were washed three times for 5 min each in PBST and 50 μl of secondary antibody [goat anti-rabbit Alexa Fluor-568 (1:200, Invitrogen, A11036) and goat anti-mouse Alexa Fluor-488 (1:200, Invitrogen, A11001)] was added to the slides. The slides were incubated in a dark humid chamber for 2 h. The slides were then washed three times and counterstained with DAPI (2 μg/ml) for 5 min, then washed once in PBST for 5 min. Then a glass coverslip was placed on top of the samples (Globe Scientific #1.5, 1404–15) with ProLong Glass (Invitrogen, P36980). Images were obtained using an LSM880 confocal microscope (Carl Zeiss, Inc) with Airyscan detector and a 63× objective. Each gonad was imaged to acquire the full Z-range of the gonad with a constant Z-step of 0.2 μm. Representative images are half Z-stack projections. Image processing and analysis was performed using Fiji (44). For samples with only DAPI staining (Fig. 1*B*), after immersion in −20 °C 100% methanol for 1 min, the slides were washed once in PBST for 5 min, incubated in the dark with DAPI (2 μg/ml) for 5 min, then washed once in PBST for 5 min. The slides were then mounted on a glass coverslip with Vectashield. Z-stack images of postmeiotic hermaphrodite sperm were obtained using a Zeiss LSM980 confocal microscope using a 40× objective lens, with 0.2 μm Z-steps. Images are Z-projections through the full Z-stack range. Thirty animals were imaged for each genotype. Image processing and analysis was performed using Fiji (44).

### Whole mount DAPI staining

L4 hermaphrodites were incubated overnight (16–24 h) at 20 °C. A 5 μl drop of M9 buffer was placed on a glass slide and 5 to 10 adult animals added to the drop. The M9 was removed by wicking with a Kimwipe. Once all M9 had been removed, 15 μl of 100% methanol was added to the animals and then allowed to evaporate. Immediately following methanol evaporation, 12 μl of 2 μg/ml DAPI was added to the animals. The animals were then covered with a coverslip, the coverslip edges sealed with nail polish, and incubated for at least 30 min prior to imaging. Imaging was done with a LSM980 microscope using a 40× objective lens, with 0.2 μm Z-steps. Images are Z-projections through the entire animal. At least ten animals were imaged for each genotype. Image processing and analysis was performed using Fiji (44).

### Whole-worm protein lysates and Western blot analysis

Six to eight 60 mm petri dishes were prepared with 10 L4 hermaphrodite worms each and allowed to grow to gravid adults of the following generation. Adult hermaphrodites from the 60 mm petri dishes were bleached to synchronize the embryos. Synchronized embryos were grown on at least 18 100 mm petri dishes until the larvae reached the L3 stage. The worms were then shifted to 24 °C for 10 to 12 h until L4 stage during which the hermaphrodite germ line is undergoing spermatogenesis. The worms were washed off the petri dishes with M9 buffer. The L4 worm pellets were flash frozen in liquid nitrogen and stored at −80 °C. Protein extraction was performed through the sequential processes of pellet grinding, resuspension in lysis buffer, sonication, and centrifugation as described in Zanin *et al.*, 2011 (38). Subsequently, 80 μg of total protein for each sample was loaded onto a 10% polyacrylamide gel (Bio-Rad Laboratories), transferred onto a 0.45 μm nitrocellulose membrane (Bio-Rad Laboratories), and blocked for 1 h in 5% milk in 1x Tris-buffered saline + 0.1% Tween 20 (TBST). The membranes were blotted for the respective proteins overnight at 4 °C. The blots were washed four times 5 min in TBST and incubated with secondary antibody for 1 h at room temperature. After incubation with secondary antibodies, the blots were washed 4 times 5 min in TBST. Proteins were detected with Clarity MAX ECL Western blotting substrate and imaged with a ChemiDoc Imaging system (Bio-Rad Laboratories). Specificity of the anti-RFP antibody was validated by Western blot. Whole worm lysates from worms expressing wrmScarlet::TDPT-1 or TDPT-1::3xFLAG were probed with anti-RFP antibody. A band corresponding to wrmScarlet::TDPT-1 (67 kDa) was detected in the wrmScarlet::TDPT-1 lysates, but not in the TDPT-1::3xFLAG lysates. Primary antibodies: anti-RFP (1:1000 in TBST, Invitrogen PA534974), anti-FLAG (1:500 in TBST, Sigma-Aldrich, F1804), anti-β-actin (loading control, 1:5000 in TBST, Abcam, AB8227). Secondary antibodies: goat anti-rabbit horseradish peroxidase (HRP)-conjugated (Invitrogen 31460) and anti-mouse HRP-conjugated (Invitrogen 31430) antibodies (1:10,000 in TBST). Three biological replicates were conducted per experiment. Analysis of Western blots was conducted and quantified using Fiji gel analysis tool (44).

### Co-immunoprecipitation experiments

Worm protein lysates were generated as described above. In a 1.5 ml tube, 50 μl of anti-FLAG magnetic agarose bead slurry (Pierce Manufacturing, A36797) was added to 450 μl of 4 °C wash buffer (10 mM Tris/Cl pH 7.5, 150 mM NaCl, 0.5 mM EDTA). The beads were washed three times by separating magnetic beads from the wash buffer with a magnetic stand. In addition, 1.5 mg of worm protein lysates were diluted with 300 μl of wash buffer and loaded onto washed magnetic beads (50 μl of lysate was reserved for “input” blot analysis). Proteins were allowed to bind with magnetic beads for 1 h with constant rotation at 4 °C. A flow-through sample was separated from the beads after the 1 h incubation. The beads were washed three times with 500 μl wash buffer. Protein elution was conducted by boiling the beads (95 °C) for 10 min in 50 μl 3× sample buffer (180 mM Tris/Cl pH 6.8, 30% glycerol, 6% SDS, 0.04% bromophenol blue, and 5% β-mercaptoethanol). The eluate was collected for Western blot. Twenty-five microliters of input and eluate of each sample was loaded onto a 7.5% polyacrylamide gel (Bio-Rad Laboratories), transferred onto a 0.45 μm nitrocellulose membrane (Bio-Rad Laboratories), and blocked for 1 h in EveryBlot blocking buffer (Bio-rad Laboratories). The membranes were incubated in primary antibody overnight at 4 °C. The blots were washed four times 5 min in TBST and incubated with secondary antibody for 1 h at room temperature. Next, the membranes were washed four times 5 min in TBST. Proteins were detected with Clarity MAX ECL Western blotting substrate and imaged with a ChemiDoc Imaging system (Bio-Rad Laboratories). When necessary, the membrane was stripped by washing the membrane two times for 15 min in stripping buffer (25 mM glycine pH 2.0, 1% SDS). After stripping, the membrane was washed for 5 min with TBST, reblocked, and continued with Western blot protocol as described above. Primary antibodies: anti-RFP (1:1000 in EveryBlot, Invitrogen, PA534974), anti-FLAG (1:500 in EveryBlot, Sigma-Aldrich, F1804), anti-SUMO (1:10 in EveryBlot, Developmental Studies Hybridoma Bank), anti-Ubiquitin (1:1000 in EveryBlot, EMD Milipore 05–944). Secondary antibodies: goat anti-rabbit HRP-conjugated (Invitrogen, 31460) and anti-mouse HRP-conjugated (Invitrogen, 31430) antibodies (1:1000 in EveryBlot). Three biological replicates were conducted per experiment. Analysis of immunoblots was conducted and quantified using ImageJ gel analysis tool (44).

### Expression of TDPT-1 proteins in *E. coli* and protein purification

#### Plasmid generation

His-SUMO-tagged *C. elegans tdpt*-1 plasmid, constructed for codon optimization in *E. coli,* was a gift from Dr Hideki Aihara (University of Minnesota). Using this WT plasmid, recombinant plasmids harboring the *tdpt*-1 point mutations were generated *via* site-directed mutagenesis in BL21 (DE3) *E. coli* for bacterial expression. QuickChange II kit (Agilent Technologies) was used for site-directed mutagenesis by following the manufacturer’s methods and instructions. Primers were designed to incorporate the *tdpt-1* point mutations as desired using the indicated primer sets (Table S3). Plasmids were submitted for Sanger sequencing (Azenta Life Sciences). Transformation of correctly mutated plasmids was performed *via* heat shock into BL21 (DE3) *E. coli* cells. All the generated plasmids were also transformed into *E. coli* DH5α cells for long term plasmid maintenance.

#### Protein expression

TDPT-1 WT and mutant protein expressing BL21 (DE3) *E. coli* cells were precultured in 10 ml of Mg containing 1.5xYT broth (1.3% tryptone, 0.75% yeast extract, 0.75% NaCl, and 0.06% MgSO_4_.7H_2_O) using appropriate antibiotics and shaking at 30 °C overnight. The saturated overnight cultures were inoculated into each 2 L flask containing 1 L of 1.5xYT+Mg broth media with antibiotics at 37 °C with horizontal rotation (250 rpm) in an incubator until absorbance values of 0.6 to 0.8 were attained. Protein expression was induced by addition of 0.4 mM isopropyl β-D-1-thiogalactopyranoside (IPTG, Thermo Fisher Scientific, 9H1805S) at 15 °C with shaking for 18 to 20 h.

#### Protein harvesting, lysis, and clarification

All the procedures were carried out at 0 to 4 °C unless stated otherwise. First, the cultures were centrifuged for 30 min at 4000 rpm. Supernatant was discarded and the remaining cell pellet was resuspended in 25 ml lysis buffer (25 mM Hepes, pH 7.5; 300 mM NaCl; 10 mM imidazole, pH 7.5, and 10% glycerol) along with addition of 1 mM phenylmethylsulfonyl fluoride (PMSF, Sigma-Aldrich, 1135906001). Resuspended pellets were either stored at −80 °C or used immediately depending on the time availability. Cells were lysed by addition of 1 mg/ml lysozyme (Thermo Fisher Scientific, J60701.14), 1 ml nuclease (Pierce universal nuclease, Thermo Fisher Scientific, J60701.14), 0.5% 3-[(3-cholamidopropyl) dimethylammonio]-1-propanesulfonate (Chaps, Thermo Fisher Scientific, 28299) and incubation on ice for 30 min, followed by sonication. About 6 to 8 rounds of sonications were performed in ice using a sonicator, allowing at least 5 min rest between rounds. The temperature of samples was maintained at or below 10 °C at the beginning of each round of sonication. For each round, a total of 30 s sonication time (5 s pulses with 10 s pauses) at 100% power was used. The lysed cell suspension was centrifuged for 1 h at 15,000*g*. The resultant supernatant was used for protein purification.

#### Protein purification

The supernatant was added to Ni^2+^-NTA agarose (MCLAB NINTA-500) preequilibrated in lysis buffer (25 mM Hepes, pH 7.5; 300 mM NaCl; 10 mM imidazole, pH 7.5; and 10% glycerol) and allowed to batch bind by rocking at 4 °C for 2 h. Resuspended beads were then allowed to flow over the column by gravity. Columns were washed with 2 column volumes of wash buffer (25 mM Hepes, pH 7.5; 300 mM NaCl; 25 mM imidazole, pH 7.5; and 10% glycerol). Elution was done into at least three fractions using an elution buffer (25 mM Hepes, pH 7.5; 300 mM NaCl; 300 mM imidazole, pH 7.5; and 10% glycerol).

Fractions containing His-SUMO-TDPT-1 proteins were analyzed by SDS-PAGE gels stained with Coomassie blue. After confirmation of desired bands (∼58 kDa), the Ni^2+^-NTA fractions were pooled and concentrated to less than 8 ml using Amicon Ultra-15 centrifugal filters with molecular weight cutoff (MWCO) of 30 kDa (Millipore Sigma, UFC9030). The concentrated proteins were dialyzed using cellulose membrane dialysis tubing with 14 kDa MWCO (Millipore Sigma) against 1 L of buffer A (25 mM Hepes, pH 7.5; 50 mM NaCl; 0.5 mM EDTA; 1 mM DTT; and 10% glycerol) at 4 °C with continuous stirring overnight. The dialyzed fraction was loaded at a flow rate of 2 ml/min into a Source15Q resin (Cytiva 17–0947–01) with a Tricorn 10/100 ion-exchange column (8 ml bed, Cytiva 28-4064-15) equilibrated with buffer A in an ÄKTA system. Then the bound proteins were eluted with a linear gradient of high salt using buffer B (25 mM Hepes, pH 7.5; 750 mM NaCl; 0.5 mM EDTA; 1 mM DTT; and 10% glycerol) and fractionated at 2 ml per tube.

SourceQ protein fractions were analyzed by SDS-PAGE, concentrated to 2.5 ml, and subjected to His-SUMO tag cleavage by incubating with Ulp-1 SUMO protease for 3 h at 25 °C. Complete cleavage was confirmed by SDS-PAGE analysis. TDPT-1 proteins were purified from the cleaved protein mixture by size-exclusion chromatography over a HiLoad 16/600 Superdex 75 pg prepacked column (Cytiva 28-9893-33). The 2.5 ml cleavage reaction protein mixture was injected into the Superdex 75 column, equilibrated with assay buffer (100 mM NaCl, 20 mM Tris pH 7.5, and 2 mM MgCl_2_) in the ÄKTA system at flow rate of 1 ml/min, and collected as 1 ml fractions per tube. Bovine γ-globulin (158 kDa), chicken ovalbumin (45 kDa), and horse myoglobin (17 kDa) were used as molecular weight standards. After SDS-PAGE analysis, the fractions containing purified TDPT-1 proteins (∼41 kDa) were pooled, concentrated using Amicon Ultra-4 centrifugal filters-MWCO 30 kDa (Millipore Sigma UFC803024), aliquoted and stored at −80° C until use.

#### Purification of Ulp-1 SUMO protease

For purification of Ulp-1 SUMO protease, all the steps were performed as described above for TDPT-1 proteins, with the following differences. Ulp-1 expression plasmid was induced for protein expression using IPTG at 0.5 mM concentration at 30 °C for 6 h. Chaps was avoided during lysis. Ion exchange chromatography was skipped and after Ni^2+^-NTA chromatography, protein was directly subjected to size exclusion using Superdex75. Instead of using the assay buffer as equilibration buffer in the Superdex75 column, Ulp-1 buffer containing 500 mM Hepes, pH 8; 500 mM NaCl, 1 mM DTT, and 0.2% octylphenoxypolyethoxyethanol was used. Equal volume of 50% glycerol was added to finally purified Ulp-1 protein before dispensing into aliquots and stored at −80 °C.

### TDPT-1 chromogenic phosphodiesterase assay

Phosphodiesterase activity was assessed *in vitro* using chemical substrates PNPP and T5PNP. PNPP has been previously demonstrated to be a minimal substrate for Tdp2, but T5PNP is a more complex substrate with an extra 5′-nucleotide, which resembles typical TDP2 substrates (5′-four nucleotide DNA overhang) (46, 47). TDP2 cleaves PNPP and T5PNP to release soluble p-nitrophenol (PNP), which is observed by a yellow color (47). The change in color was detected as an increase in absorbance at 415 nm using a Tecan Spark multimode microplate reader. The *in vitro* assay was performed in 384-well clear plates with triplicate samples in reaction buffer containing 100 mM NaCl, 2 mM MgCl_2_, 20 mM Tris, pH 7.5 and 0.1 μM purified TDPT-1 proteins. The reaction was initiated by addition of 10 mM chemical substrates, which was followed by immediate absorbance signal acquisition in the Tecan Spark multimode microplate reader. Absorbance monitoring at 415 nm was performed for 60 min for the T5PNP while PNPP required a longer time period (360 min). Data were analyzed and plotted using GraphPad Prism 9.4.1 (https://graphpad.com/).

### Thermal shift assay

#### Experimental set up and conditions

Thermal shift assays were performed using a QuantStudio 6 Flex Real-Time PCR system. SYPRO Orange (Sigma-Aldrich, S5692) was used at a final concentration of 20× in 50 μl reactions. Reactions were carried out on ice using MicroAmp optical 96-well reaction plates (Thermo Fisher Scientific, N8010560) in three 50 μl replicates for each sample. Each reaction well was dispensed in order with an assay buffer containing 100 mM NaCl, 2 mM MgCl_2_, 20 mM Tris, pH 7.5 followed by protein samples and the dye. Buffer alone was used as negative control. Sumo protease Ulp-1 and chicken egg lysozyme (Alfa Aesar J60701) were used as positive controls. The control proteins were used at a concentration of 0.5 μg/μl and the TDPT-1 proteins were used at a concentration of 5 μg/μl. The reaction plates were sealed with MicroAmp optical adhesive film (Thermo Fisher Scientific, 4313663) and centrifuged at 4000*g* for 5 min at 4 °C. Using the melting curve method, the plates were heated from 15 to 99 °C at a rate of 1 °C/min and fluorescence was collected using SYBR reporter, no quencher, and no passive reference.

#### Estimation of Tm

Melting curve fluorescence values were exported from the instrument in excel files. The dataset included 25 data points after the maximum fluorescence value. The normalized fluorescence data as a function of temperature were fitted to nonlinear curve fit to Boltzmann Sigmoidal model in GraphPad Prism 9.4.1. Then, the first derivative of the nonlinear fit of data was obtained to evaluate the Tm. The Boltzmann sigmoid uses the equation:

$$F=F_{\min}+\left(F_{\max}\;-\;F_{\min}\right)/\left(1+\exp\left(Tm\;-\;T/slope\;at\;Tm\right)\right)$$

where F= fluorescence emission at temperature T, F_min_ = baseline fluorescence at low temperature, F_max_ = maximum fluorescence and Tm = melting temperature of the protein.

## Data availability

All data are included in the manuscript and in the supporting information.

## Supporting information

This article contains supporting information (3, 12, 24).

## Conflict of interest

The authors declare that they have no conflicts of interest with the contents of this article.
