# Supplementary material for: Mutations in tyrosyl-DNA phosphodiesterase 2 suppress top-2 induced chromosome segregation defects during Caenorhabditis elegans spermatogenesis
Source: J Biol Chem. 2024 Jun 4;300(7):107446. doi: 10.1016/j.jbc.2024.107446 (PMC11261448; doi:10.1016/j.jbc.2024.107446)
Supplement: Supporting Information [file mmc1.pdf]

Mutations in Tyrosyl-DNA Phosphodiesterase 2 suppress *top-2* induced chromosome segregation defects during *C. elegans* spermatogenesis

Ji Kent Kwah[1*], Nirajan Bhandari[1,2*], Christine Rourke[1], Gabriella Gassaway[1,3] and Aimee Jaramillo-Lambert[1§]

Supporting Information:

Figure S1: *top-2* and *tdpt-1* brood sizes.

Figure S2. Homozygous *top-2* [R828C]::*gfp* hermaphrodites have meiotic defects and disrupted germlines.

Figure S3. *top-2* [G117R] rescues *top-2* [R828C] embryonic lethality.

Figure S4. SDS-PAGE analysis of TDPT-1 proteins.

Figure S5. TDPT-1 does not appear to be modified by ubiquitination or SUMOylation.

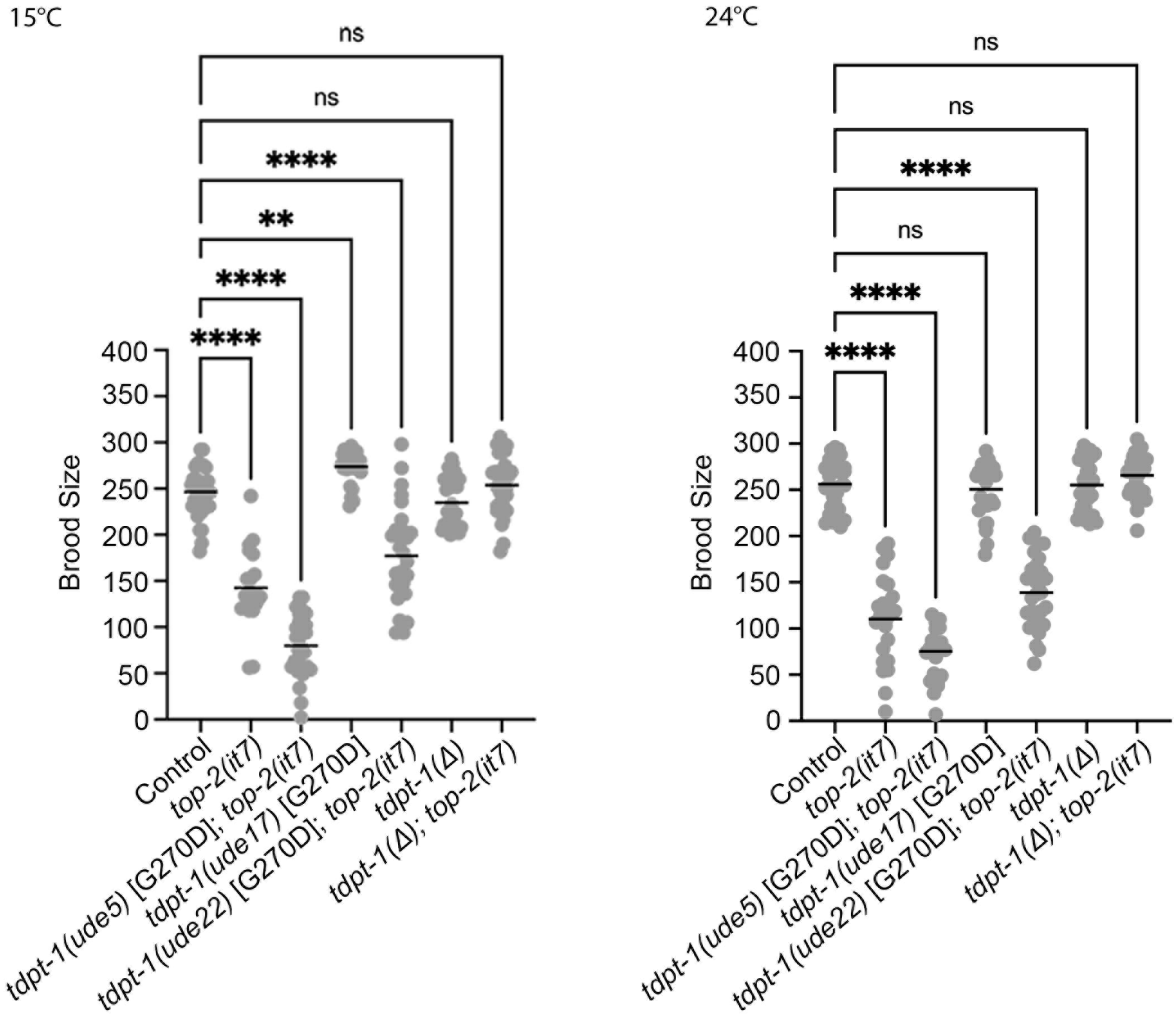

Figure S1. *top-2* and *tdpt-1* brood sizes. Brood size counts for control [N2], *unc-4(e120) top-2(it7)*, *tdpt-1(ude5)* [G270D]; *unc-4(e120) top-2(it7)*, *tdpt-1(ude17)* [G270D], *tdpt-1(ude22)* [G270D]; *unc-4(e120) top-2(it7)*, *tdpt-1(tn1426Δ)*, and *tdpt-1(tn1526Δ); unc-4(e120) top-2(it7)* at 15°C (left) and 24°C (right). The brood of each individual hermaphrodite was plotted from three replicate experiments (5-10 worms each replicate for a total of 20-30 worms per strain). The mean brood size is indicated by the black bar. At least 2212 progeny (dead embryos and live larvae) were scored for each genotype and temperature. P-values were calculated by one-way ANOVA. ****p<0.0001, **p<0.05.

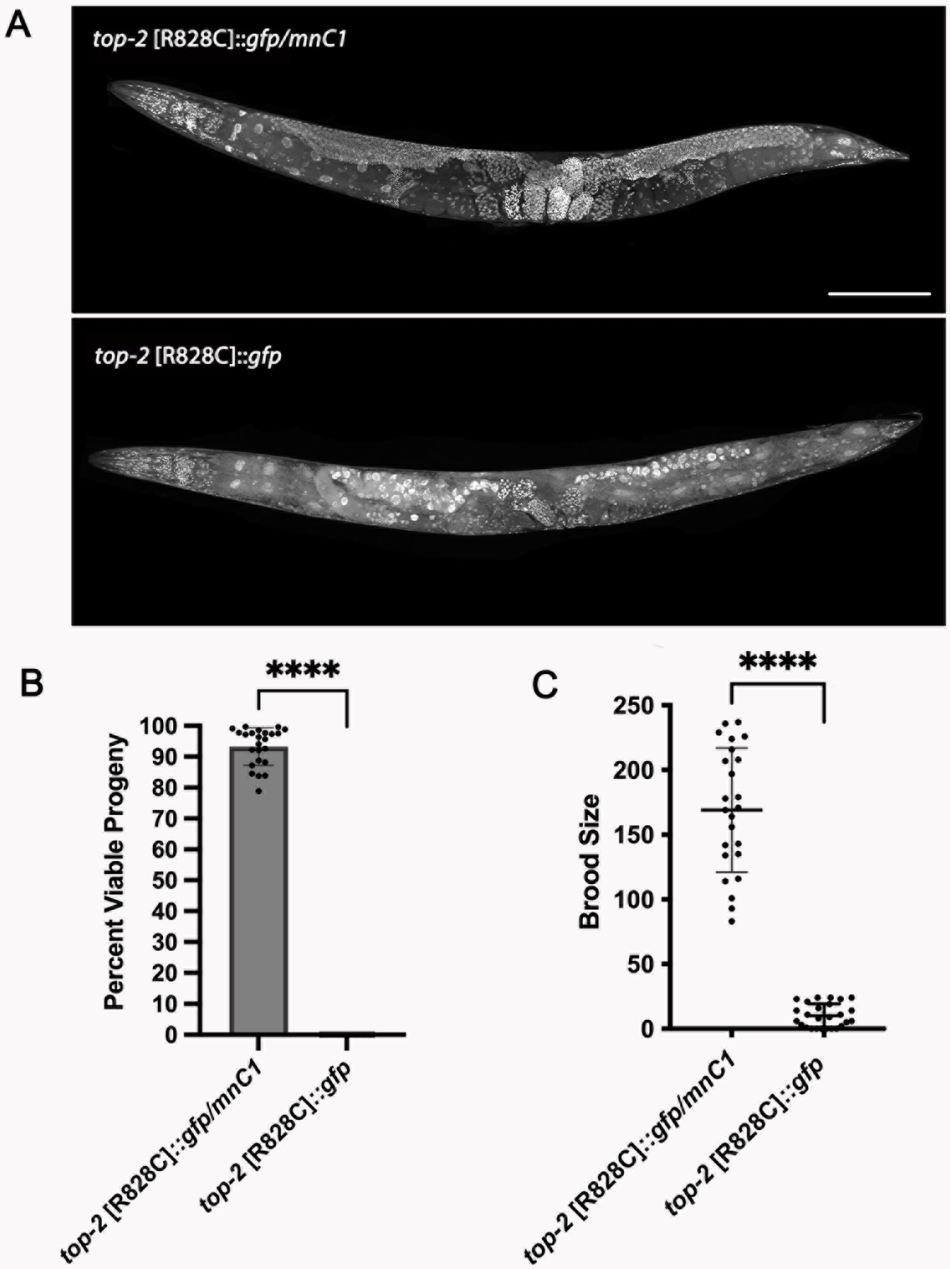

Figure S2. Homozygous top-2 [R828C]::gfp hermaphrodites have meiotic defects and disrupted germlines. (A) Whole worm DAPI staining of *top-2* [R828C]*::gfp/mnC1* (top) and homozygous *top-2* [R828C]*::gfp* (bottom) hermaphrodites. Worms were fixed and stained 24 h after the L4 stage. Scale bar = 100 µm. (B) Graph depicting percent viable progeny for individual *top-2* [R828C]*::gfp/mnC1* and homozygous *top-2* [R828C]*::gfp* hermaphrodites at 20°C. (C) Graph depicting brood size for individual hermaphrodites for *top-2* [R828C]*::gfp/mnC1* and homozygous *top-2* [R828C]*::gfp* hermaphrodites at 20°C. Error bars represent SD. ****= p<0.0001.

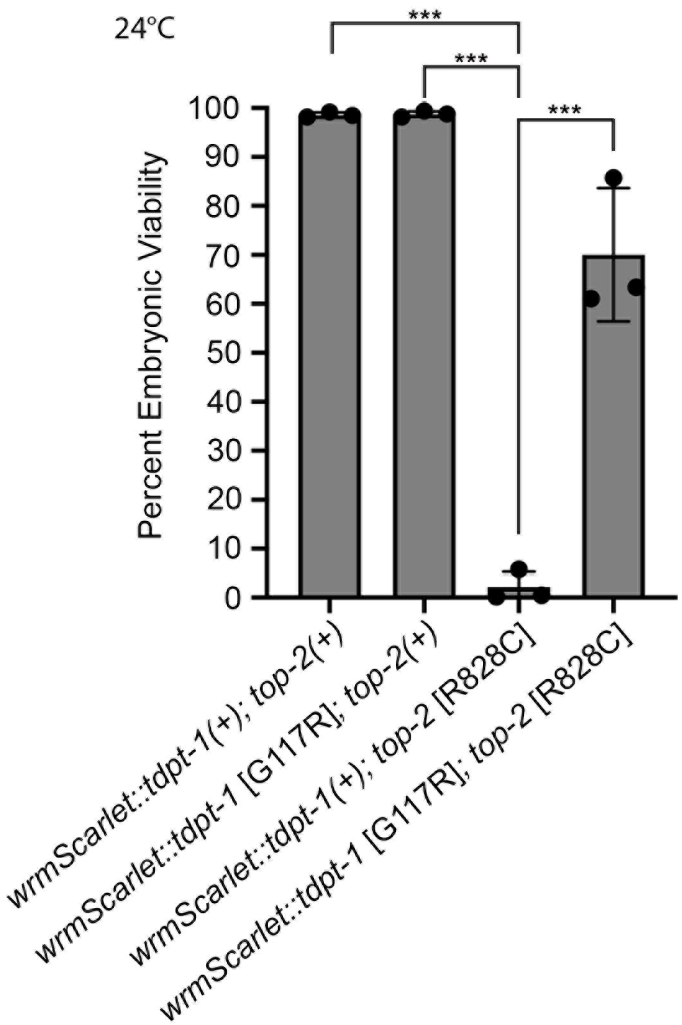

Figure S3. *top-2* [G117R] rescues *top-2* [R828C] embryonic lethality. Percent embryonic viability from *unc-4(e120) top-2(it7)*, *wrmScarlet::tdpt-1; top-2(it7); him-8(e1489)*, *wrmScarlet::tdpt-1*, *wrmScarlet::tdpt-1* [G117R], and *wrmScarlet::tdpt-1* [G117R]; *unc-4(e120) top-2(it7)* at 24°C. The data is represented as the average of three individual replicate experiments (8-10 worms each replicate for a total of 28-30 worms per strain) with error bars representing SEM. At least 4120 progeny were scored for each genotype. P-values were calculated using a Cochran-Mantel-Haenszel test with Bonferroni correction (0.008). Any value <0.008 is statistically significant.

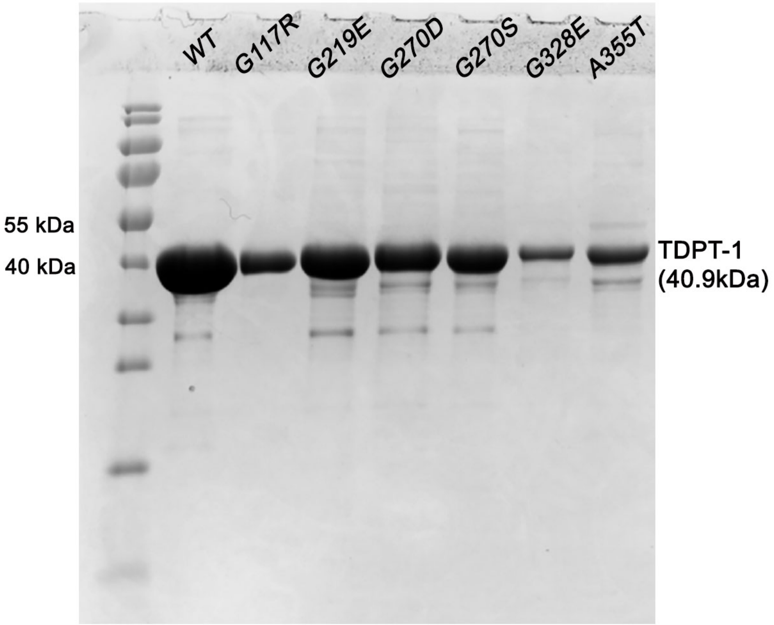

Figure S4. SDS-PAGE analysis of TDPT-1 proteins. Purified TDPT-1 proteins were analyzed by SDS-PAGE and stained with Coomassie blue. Concentrated Ulp-1 cleaved protein mixtures were subjected to SEC on a Superdex75 column for final purification. Aliquots from final purifications of WT, G117R, G219E, G270D, G270S, G328E and A355T TDPT-1 proteins were loaded in lanes as shown. All TDPT-1 proteins can be observed at the expected molecular weight of ~41 kDa.

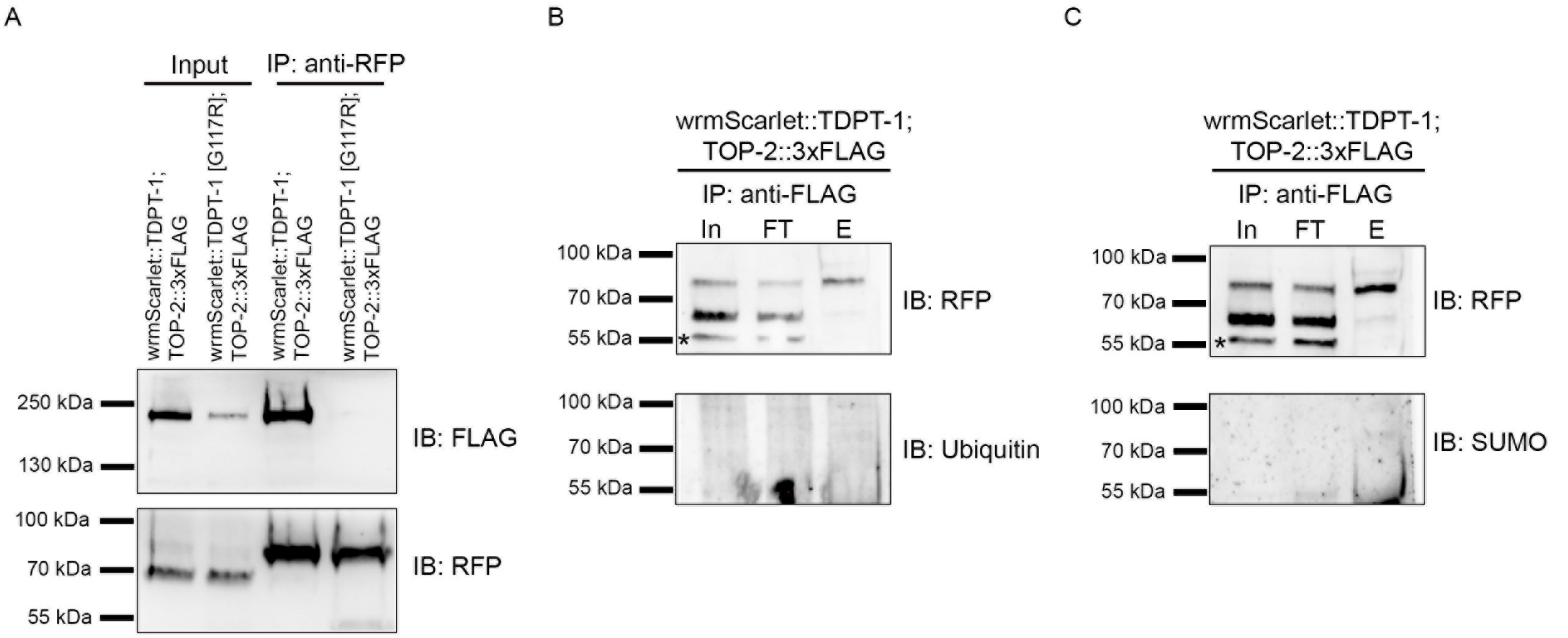

Figure S5. TDPT-1 does not appear to be modified by ubiquitination or SUMOylation. (A) Co-IP of wrmScarlet::TDPT-1 with TOP-2::3xFLAG. (B-C) Western blot to determine if wrmScarlet::TDPT-1 post-translational modification was ubiquitination (B) or SUMOylation (C).These membranes were first immunoblotted with either anti-ubiquitin or anti-SUMO, then stripped and the membranes immunoblotted for wrmScarlet::TDPT-1 (IB: RFP). *Notes a non-specific band that appears in the anti-RFP blots only after stripping and re-immunoblotting.
