# Supplementary material for: Mutations in tyrosyl-DNA phosphodiesterase 2 suppress top-2 induced chromosome segregation defects during Caenorhabditis elegans spermatogenesis
Source: J Biol Chem. 2024 Jun 4;300(7):107446. doi: 10.1016/j.jbc.2024.107446 (PMC11261448; doi:10.1016/j.jbc.2024.107446)
Supplement: Table S1 [file mmc2.docx]

**Table S1. Strains used in this study.**

| **Strain Name** | **Genotype** | **Citation/**  **Source** |
| --- | --- | --- |
| **20°C** |  |  |
| N2 | wild-type Bristol isolate | CGC |
| DG3833 | *tdpt-1(tn1526∆ [loxP unc-119(+) loxP])* I;  *unc-119(ed3)* II | David Greenstein  UMN |
| PHX2976 | *wrmScarlet::tdpt-1(syb2976)* I | Bhandari et. al. 2022 |
| AJL22 | *tdpt-1(ude17)* [CRISPR recreate of *ude5* in N2] I | Bhandari et. al. 2020 |
| AJL37 | *tdpt-1(syb2976)* [*wrmScarlet::tdpt-1(+)*] I; *top-2(av87)* [*top-2(+)::gfp*] II | This study |
| AJL45 | *tdpt-1(syb2976)* [*wrmScarlet::tdpt-1(+)*] I; *top-2(av64)* [*top-2(+)::3xflag*] II | This study |
| AJL83 | *tdpt-1(ude41)* [*wrmScarlet::tdpt-1* (G117R)] I;  *top-2(av77)* [*top-2* [R828C]*::3xflag*] II | This study |
| AJL96 | *tdpt-1(ude43)* [*wrmScarlet::tdpt-1* (G270D)] I;  *top-2(av87)* [*top-2(+)::gfp*] II | This study |
| AJL101 | *top-2(ude45)* [*top-2* (R828C)*::gfp*]*/mIn1* | This study |
| AJL104 | *tdpt-1(ude46)* [*wrmScarlet::tdpt-1*(G270D)] I;  *top-2(av77)* [*top-2(+)::3xflag*] II | This study |
| AJL129 | *tdpt-1(ude41)* [*wrmScarlet::tdpt-1* (G117R)] I | This study |
| **15°C** |  |  |
| KK381 | *unc-4(e120) top-2(it7)* II | CGC |
| AJL5 | *tdpt-1(ude5)* [G270D] I; *unc-4(e120) top-2(it7ts)* II | Bhandari et. al. 2020 |
| AJL27 | *tdpt-1(ude22)* [CRISPR recreate of G270D] I;  *unc-4(e120) top-2(it7ts)* II | Bhandari et. al. 2020 |
| AJL36 | *tdpt-1(tn1526∆ [loxP unc-119(+) loxP])* I; *unc-4(e120) top-2(it7)* II | This study |
| AJL43 | *tdpt-1(syb2976)* [*wrmScarlet::tdpt-1(+)*] I; *top-2(it7)* II; *him-8(e1489)* IV | This study |
| AJL78 | *tdpt-1(syb2976)* [*wrmScarlet::tdpt-1(+)*] I; *top-2(av77)* [*top-2* (R828C)*::3xflag*] II | This study |
| AJL86 | *tdpt-1(ude43)* [*wrmScarlet::tdpt-1* (G270D)] I; *top-2(it7)* II; *him-8(e1489)* IV | This study |
| AJL105 | *tdpt-1(ude41)* [*wrmScarlet::tdpt-1* (G117R)] I; *top-2(av77)* [*top-2* (R828C)*::3xflag*] II | This study |
| AJL138 | *tdpt-1(ude41)* [*wrmScarlet::tdpt-1* (G117R)] I; *unc-4(e120) top-2(it7)* II | This study |
