# Supplementary material for: Mutations in tyrosyl-DNA phosphodiesterase 2 suppress top-2 induced chromosome segregation defects during Caenorhabditis elegans spermatogenesis
Source: J Biol Chem. 2024 Jun 4;300(7):107446. doi: 10.1016/j.jbc.2024.107446 (PMC11261448; doi:10.1016/j.jbc.2024.107446)
Supplement: Table S2 [file mmc3.docx]

**Table S2. crRNAs and repair templates for mutant alleles generated in this study.**

| **Allele(s)** | **crRNAs** | **Repair Template** | **Citation**  (when used in previous study) |
| --- | --- | --- | --- |
| *tdpt-1*  [G270D] | 5’- CCGGGC  GCCCTCGTC  TTTTT -3’ | 5’- GTCCGTGAAATCATCGCTCAAAACCCGG  GCGCCCT*G*GT*G*TT*C*TT*T*GGCG**A**CGATTTAAA  CTTACGAGACGAGGAGGTCAGCCGTGTGCC  TGACG -3’ | Bhandari et al 2020 |
| *tdp-t-1*  [G117R] | 5’- GATGACG  GCAGAGGAT  TTGA -3’ | 5’- tttttcgcgatttttccaacaaatttcaaatttttgaag**A**GATTCG  AAGTCAGCGTAATGTCGTGGAACATTGAT -3’ | This study |
| *top-2* [R828C] | 5’-CTTCTTCC  AATCGGACA  GTT -3’ | 5’- CTCGCTCAAGATTACGTTGGCTCCAACAACA  TCAACCTGCTTCTTCCAAT*A*GG*G*CA*A*TT*C*GGTA  CT**T**GTCTGCAGGGTGGAAAGGACAGTGCTTC AGCTCGTTACATCTTCACTCAACTGTCGCC -3’ | Jaramillo-Lambert et al 2016 |

In the repair template sequences, uppercase letters are exonic sequences and lowercase are intronic sequences. Bolded letters are the edited nucleotides and restriction enzyme recognition sites are underlined. Mutations to prevent Cas9 cleavage after incorporation of the repair template are indicated with italics.
