# Supplementary material for: Mutations in tyrosyl-DNA phosphodiesterase 2 suppress top-2 induced chromosome segregation defects during Caenorhabditis elegans spermatogenesis
Source: J Biol Chem. 2024 Jun 4;300(7):107446. doi: 10.1016/j.jbc.2024.107446 (PMC11261448; doi:10.1016/j.jbc.2024.107446)
Supplement: Table S3 [file mmc4.docx]

**Table S3: Primers used for site directed mutagenesis.**

| TDPT-1 mutation (codon change) | Primers | Nucleotide sequence |
| --- | --- | --- |
| G117R  (GGT>CGT) | G117R_opti_F | 5'-cgctgaagacctgaag**cgt**ttcgaagtttccgt-3' |
|  | G117R_opti_R | 5'-acggaaacttcgaaacgcttcaggtcttcagcg-3' |
| G219E  (GGG>GAG) | G219E_opti_F | 5'-tgcagatcctggag**gag**tctatcggtggcctg-3' |
|  | G219E_opti_R | 5'-caggccaccgatagactcctccaggatctgca-3' |
| G270D  (GGT>GAT) | G270D_opti_F | 5'- ctggtgttcttcggc**gat**gatctgaatctgcgc-3' |
|  | G270D_opti_R | 5'-gcgcagattcagatcatcgccgaagaacaccag-3' |
| G270S  (GGT>AGT) | G270S_opti_F | 5'-gctggtgttcttcggc**agt**gatctgaatctgcg-3' |
|  | G270S_opti_R | 5'-cgcagattcagatcactgccgaagaacaccagc-3' |
| G328E  (GGC>GAG) | G328E_opti_F | 5'-cgcctgtactggtct**gag**ccactggacaaagtga-3' |
|  | G328E_opti_R | 5'-tcactttgtccagtggctcagaccagtacaggcg-3' |
| A355T  (GCT>ACT) | A355T_opti_F | 5'-ccgtccgaccattgg**act**atcaacgcaacgt-3' |
|  | A355T_opti_R | 5'-acgttgcgttgatagtccaatggtcggacgg-3' |

Primers were designed for amplifying the TDPT-1 plasmid with the desired mutation. Codon changes are bolded. F and R denote forward and reverse primers respectively.
